# Efficacy and Safety of Auricular Acupuncture for Depression: A Randomized Clinical Trial

**DOI:** 10.1001/jamanetworkopen.2023.45138

**Published:** 2023-11-30

**Authors:** Daniel Maurício de Oliveira Rodrigues, Paulo Rossi Menezes, Ana Elise Machado Ribeiro Silotto, Artur Heps, Nathália Martins Pereira Sanches, Mariana Cabral Schveitzer, Alexandre Faisal-Cury

**Affiliations:** 1 Department of Preventive Medicine, Faculdade de Medicina, Universidade de São Paulo, São Paulo, Brazil; 2 Department of Biological and Health Sciences, University of Southern Santa Catarina, Palhoça, Brazil; 3 Department of Health Sciences, Cruzeiro do Sul University, São Paulo, Brazil; 4 Institute of Psychiatry of Santa Catarina, São José, Santa Catarina, Brazil; 5 Department of Preventive Medicine, Escola Paulista de Medicina, Universidade Federal de São Paulo, São Paulo, Brazil

## Abstract

**Importance:**

Depression is a leading cause of disability worldwide, and there is increasing interest in nonpharmacological treatments. Auricular acupuncture (AA) is a simple, low-cost, and well-tolerated option, but further studies are needed to establish its efficacy and safety.

**Objective:**

To estimate the efficacy and safety of auricular acupuncture as a treatment for depression.

**Design, Setting, and Participants:**

This randomized clinical trial was conducted at 4 university research centers in Brazil, from March to July 2023. Eligible patients were adults aged 18 to 50 years whose score on the Patient Health Questionnaire–9 (PHQ-9) indicated moderate depression (score 10-14) or moderately severe depression (score 15-19). Exclusion criteria included previous application of AA, risk of suicidal ideation, or severe depression (PHQ-9 score >20). An intent-to-treat analysis and modified intent-to-treat analysis were conducted.

**Intervention:**

Participants were randomized into 2 treatment groups, which included specific AA (SA) and nonspecific AA (NSA). Both groups received 12 sessions of AA with semipermanent needles with daily stimulation twice a week over 6 weeks and were followed-up for 3 months. All participants continued with their usual care for ethical reasons. The SA group’s treatment protocol consisted of 6 acupuncture points on the auricular pavilion chosen according to the diagnosis of depression by traditional Chinese medicine (Shenmen, subcortex, heart, lung, liver, and kidney). The NSA group’s acupuncture points were the external ear, the cheek and face area, and 4 nonspecific points in the helix region unassociated with mental health symptoms. A locator device was used to confirm which areas had neuroreactive points.

**Main Outcomes and Measures:**

The primary outcome was a reduction of at least 50% in the PHQ-9 score (ie, depression recovery) at 3 months. Secondary outcomes included depression recovery at 4 and 6 weeks; depression remission (PHQ-9 score < 5) at 4 weeks, 6 weeks, and 3 months); and adverse events.

**Results:**

A total of 304 participants were screened, and 74 participants (62 women [84%]; median [IQR] age, 29 [23-27] years) were included in the intention-to-treat analysis, with 37 participants randomized to each group (SA and NSA). A total of 47 participants (64%) were followed-up through 3 months. The results showed no statistically significant difference in depressive recovery between the groups at 3 months (14 of 24 participants in the SA group [58%] vs 10 of 23 participants in the NSA group [43%]; risk ratio [RR], 1.34; 95% CI, 0.76-2.45; *P* = .38). The proportions of depression recovery and remission at 4 and 6 weeks based on the PHQ-9 were higher in the SA group (except for depression recovery at 6 weeks) with no statistically significant differences. However, a statistically significant difference was observed in symptom remission at 3 months (11 of 24 participants in the SA group [46%] vs 3 of 23 participants in the NSA group [13%]; RR, 1.99; 95% CI, 1.16-3.34; *P* = .02) in favor of SA. There were no significant differences in adverse event rates between the groups, evidencing the intervention’s safety. Most participants reported mild pain at the needle application site (33 patients [94%] in the SA group vs 32 patients [91%] in the NSA group). Five participants dropped out of the study due to adverse events.

**Conclusions and Relevance:**

The results of this randomized clinical trial suggest that SA over 6 weeks is safe. Although there was no statistically significant difference between groups for the primary efficacy outcome, patients receiving SA did experience greater symptom remission at 3 months. A larger sample size and longer intervention are needed to further evaluate the efficacy of SA for depression.

**Trial Registration:**

ClinicalTrials.gov Identifier: NCT05855421

## Key Points

**Question:**

Is auricular acupuncture a safe and effective treatment for depression?

**Findings:**

This randomized clinical trial of 74 patients with depression undergoing 6 weeks of auricular acupuncture found no significant differences in adverse event rates between the intervention (specific auricular acupuncture) and control (nonspecific auricular acupuncture) groups, evidencing the intervention’s safety. No significant differences were found in depression recovery (50% reduction in Patient Health Questionnaire–9 score from baseline).

**Meaning:**

These findings suggest that auricular acupuncture is safe for patients with depression, but additional studies with larger samples and longer interventions are needed for confirmation.

## Teaser

This randomized clinical trial analyzes the safety and efficacy of auricular acupuncture as a treatment for depression.

## Introduction

Depression is one of the leading causes of disability worldwide, affecting more than 300 million people (4.4% of the global population).^1^ In 2019, depressive disorders represented the highest proportion of disability-adjusted life-years among mental disorders (37.3%).^2^ In Brazil, the prevalence depression is among of the highest in the world, affecting 5.8% of the Brazilian population and accounting for 10.3% of years of life lost by Brazilians.^3^ However, less than 10% of those affected by the disease receive appropriate treatment.^1,4^ Nonadherence to antidepressant treatment is a concern for health care professionals; adherence to antidepressant therapy ranges from 40% to 90% across different studies, with a mean adherence rate of 65%.^5^

Research has shown that Australians and North Americans prefer integrative treatments for depression.^6,7,8^ In this sense, auricular acupuncture (AA), a type of acupuncture in which thin semipermanent needles are inserted into specific points on the auricular pavilion, may be a therapeutic option. AA is easier to implement in clinical settings than body acupuncture owing to its short application time, low technique complexity, relative safety, and daily continuous physiological stimulation.^9^

Several nerves are distributed in the auricular pavilions. The concha area has a rich distribution of the vagus nerve.^10^ Anatomical studies have shown that the auricular pavilion is the only place on the human body’s surface with a distribution of the afferent vagus nerve.^11^ Thus, needle stimulation on the auricular pavilion appears to lead to specific activation in the brain, mainly through the auricular branch of the vagus nerve.^12,13^ A systematic review^14^ found little evidence of the efficacy of AA for depression. The methodological quality scores showed that most analyzed studies indicated considerable methodological flaws. Additionally, no study in the review^14^ used semipermanent needles in the groups. Therefore, this study aims to estimate the efficacy and safety of AA for depression recovery and remission at 4 weeks, 6 weeks, and 3 months.

## Methods

### Study Design

This randomized clinical trial was approved by the Hospital das Clínicas ethics committee, the University of São Paulo, and the University of Southern Santa Catarina and was monitored by an independent data and safety monitoring board. We followed the Consolidated Standards of Reporting Trials (CONSORT) reporting guideline and the Standards for Reporting Interventions in Clinical Trials of Acupuncture (STRICTA)^15^ for the design and reporting of this study. We recruited patients from the community at 4 university research centers in Santa Catarina, Brazil, from March to April 2023. All patients provided written informed consent before participation, and participants did not receive incentives or compensation to be part of the study. The study protocol and statistical analysis plan can be found in Supplement 1.

### Participants

The research participants were adults aged 18 to 50 years. Eligible participants were those whose score on the Patient Health Questionnaire–9 (PHQ-9)^16^ indicated moderate depression (score of 10-14) or moderately severe depression (score of 15-19). Exclusion criteria included previous application of AA, risk of suicidal ideation, or severe depression (PHQ-9 score ≥20) (eTable 1 in Supplement 2). Demographic characteristics, including gender and race and ethnicity, were collected via self-report on electronic tablets using the REDCap platform. Race and ethnicity categories included Black, White, and multiracial; race and ethnicity were included in this study to align with current research.

### Randomization and Blinding

A computer program performed block randomization in a 1:1 ratio, in different block sizes (4, 6, and 8), corresponding to the 2 study groups. The experimental group received specific AA (SA) for depression and usual care, and the control group received nonspecific AA (NSA) and usual care. All participants continued with their usual care for ethical reasons. Participant allocation was concealed and delivered to the acupuncturists in individual opaque sealed envelopes. Participants, evaluators, data managers, and the statistician were blinded to treatment assignment. Neither the study participants nor the investigators had any influence on randomization and allocation concealment, which an independent statistician performed. Participants were informed that there were 2 treatment protocols.

### Procedures

The study assessors were trained to approach participants similarly to avoid recruitment biases. The study used a protocol of questions and procedures for all phases: prescreening, screening, data collection, and application of protocols.

Participants with severe depression levels (PHQ score ≥20) or suicidal ideation did not participate in the study and were followed and consulted by a psychiatrist. The sessions of AA application occurred twice weekly, alternating the application on the right and left auricular pavilions, over 6 weeks, totaling 12 sessions, with a duration of 15 minutes each, in reserved rooms. The acupuncturists instructed the participants to stimulate each point manually (de qi stimulation) 3 times a day (morning, afternoon, and evening) for 30 seconds or until the auricular pavilion became red or sensitive to pressure. In this study, the acupuncturists had specific training in the field, with a minimum of 1200 hours in acupuncture and a minimum of 10 years of experience. They received standardized training before the study began.

All participants answered the PHQ-9 questionnaires 4 weeks, 6 weeks, and 3 months after the study initiation and reported adverse events before each AA session (eFigure 1 in Supplement 2). Patients were asked to complete the blinding assessment and treatment efficacy perception assessment and were requested to guess which treatment they received to test the success of blinding after 3 months of the study. More details about AA procedures and outcome assessment can be found in Supplement 1.

### SA Treatment

The SA group’s treatment consisted of a protocol of acupuncture points chosen according to the diagnosis of depression by traditional Chinese medicine. All SA participants used 6 preestablished points on the auricular pavilion: Shenmen, subcortex, heart, lung, liver, and kidney^17,18^ (eFigure 2 and eTable 2 in Supplement 2). The EL11 device (NKL) was used to find the exact location of the points; the device searches on the skin for areas of lower electrical resistance, indicating more neuroreactive points (ie, true acupuncture points).^19^ The size of the semipermanent needle (Complementar) used was 0.2 mm wide and 2.5 mm long. The semipermanent needles were inserted to a depth of 2.5 mm.

### NSA Treatment

The existing literature acknowledges the difficulty in establishing protocols to be used as a control because of the high responsiveness and innervation of the auricular pavilion (ie, sham needling is not physiologically inert).^19,20,21^ As a control group strategy, our study opted for superficial needling at nonpoints or irrelevant true points, aiming to reach other neural segments. The NSA group points were the external ear, the cheek and face area, and 4 nonspecific points in the helix region (eFigure 3 in Supplement 2), which are points that are not associated with mental health symptoms (eTable 2 in Supplement 2). The EL11 locator device was used to confirm that the sham areas were not neuroreactive points. The size of the needle (Complementar) used in the control group was 0.2 mm wide and 1.0 mm long, aiming for more superficial needling. The semipermanent needles were inserted to a depth of 1.0 mm.

### Outcomes

The primary outcome was the proportion of participants who showed improvement of 50% or more in depressive symptoms (ie, depression recovery) between the groups, as assessed by the PHQ-9 at 3 months. Secondary outcomes included the difference in the proportion of participants reaching depression recovery at 4 and 6 weeks; the proportion of participants who achieved a score of less than 5 on the PHQ-9 (indicating depression remission) and differences in depression scores at 4 weeks, 6 weeks, and 3 months; the difference in the proportion of reported adverse events (ie, those arising from the AA technique) and reported adverse effects (ie, the worsening of depression symptoms or the onset of suicidal ideation); and the difference in the proportion of patient blinding to randomization and perception of treatment efficacy (eTable 3 in Supplement 2).

### Statistical Analysis

Data analysis in this clinical trial was conducted by an independent hired statistician, blinded to treatment groups. The sample size was designed to detect a 30% difference (experimental group, 60% and control group, 30%) in depression symptom recovery between the 2 groups. A minimum sample of 36 participants per group was estimated, considering the test as 2-tailed, with 80% power and a significance level of *P* < .05. An additional 10% was added, considering segment losses, totaling 40 participants in each group, for 80 participants.

Initially, a baseline comparison between the SA and NSA groups was performed with median and IQR descriptions for continuous variables and frequencies and percentages for categorical variables. Subsequently, intent-to-treat and modified intent-to-treat analyses were conducted to compare the proportions of depression recovery and remission at different time points in the study using Fisher exact test. The effect size for dichotomous variables was calculated as relative risk.

Scores were subjected to a normality test that rejected the normality hypothesis to verify sample normality. Therefore, nonparametric tests were adopted. The reduction of depression scores over time in both groups was evaluated using the median and IQR. The statistical analysis of these data was conducted using the Wilcoxon-Mann-Whitney *U* test.

Cochran *Q* and Friedman analysis of variance tests were also used to verify statistically significant differences in depression scores over time within each group. These tests were followed by post hoc tests to identify significant differences at specific time points. We performed a sensitivity analysis with missing data imputation to evaluate the robustness of the results. Multiple imputation methods were explored, including last observation carried forward, imputation using the generalized estimating equations model, and multiple imputation through the expectation-maximization lag algorithm for categorical variables; the generalized estimating equations model was applied specifically to PHQ-9 depression scores. All analyses were conducted with a significance level of *P* < .05. Relative and absolute frequency were used to assess treatment safety and identify adverse events. The success of blinding and the perception of treatment efficacy were assessed using Fisher exact test. Statistical analyses were performed using R statistical software version 4.1.0 (R Project for Statistical Computing).

## Results

**Figure 1.**
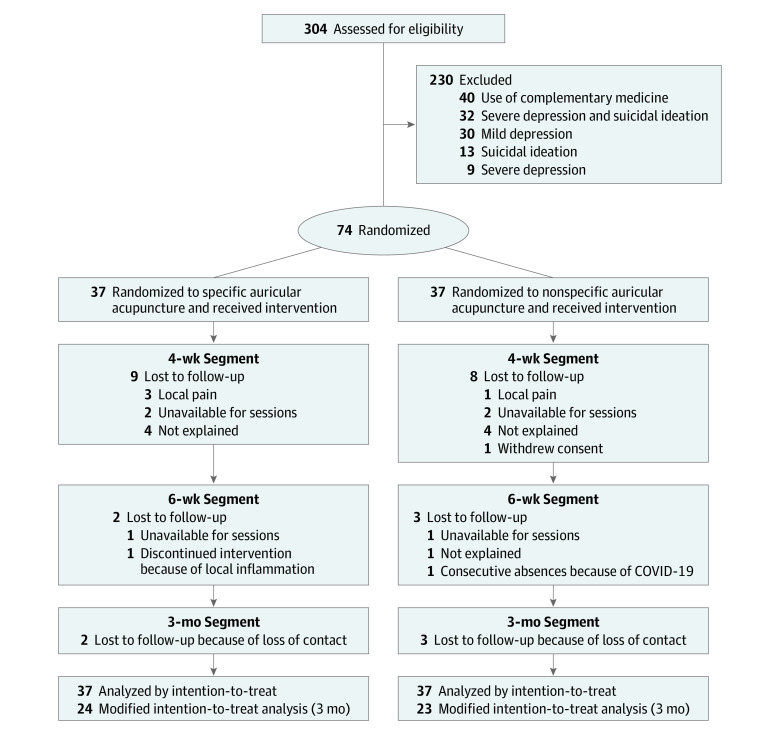
Flowchart of Screening, Randomization, and Follow-Up

Of the 304 screened volunteers, 230 were excluded for various reasons, resulting in a cohort of 74 participants (62 women [84%]; median [IQR] age, 29 [23-37] years; 5 Black participants [7%]; 61 White participants [82%]; 8 multiracial participants [10%]) comprising the intent-to-treat sample with 37 participants randomized to SA treatment and 37 participants randomized to NSA treatment (Figure 1). At 3 months, 27 patients (36%) were lost to follow-up; a total of 47 patients (64%), including 24 patients in the SA group and 23 participants, in the NSA group, comprised the modified intent-to-treat sample. There was no difference between the study groups in the number of patients lost to follow-up (eTable 4 in Supplement 2). We conducted a comparison of baseline depression scores according to the PHQ-9 among the individuals lost to follow-up (eTable 5 in Supplement 2). The groups were similar regarding demographic characteristics, clinical diagnosis, pharmacological treatments, and health characteristics at the study’s outset, except for race and ethnicity (Table 1).

**Table 1.** Baseline Demographic and Clinical Characteristics at the Time of Randomization in the Intention-to-Treat Analysis

Variable	Participants, No. (%) (N = 74)	
	SA group (n = 37)	NSA group (n = 37)
Age, median (IQR), y	28 (22-37)	30 (23-36)
Patient Health Questionnaire–9 Score, median (IQR)^a^	14.0 (12.0-16.0)	16.0 (14.0-18.0)
Gender		
Male	5 (13)	6 (16)
Female	32 (87)	30 (81)
Nonbinary	0	1 (3)
Race and ethnicity		
Black	0	5 (14)
Multiracial	2 (5)	6 (16)
White	35 (95)	26 (70)
Income^b^		
≤3 times the monthly minimum wage	14 (38)	11 (30)
>3 times the monthly minimum wage	23 (62)	26 (70)
Marital status		
Single	22 (59)	17 (46)
Not single	15 (41)	20 (54)
Education		
Less than high school up to completed high school	10 (27)	10 (27)
Incomplete college education or higher	27 (73)	27 (73)
Weekly working hours, median (IQR) (n = 49)	42 (27-44)	44 (22-44)
Previous diagnosis of depression or other mental illness	16 (43)	16 (43)
Participant used any medication for depression	8 (22)	4 (11)
Participant used medication in the last 3 mo		
No	21 (57)	27 (73)
Antidepressant	8 (22)	5 (13)
Tranquilizer or sedative	5 (13)	4 (11)
Anxiolytic	7 (19)	3 (8)
Participant consumes alcoholic beverages	26 (70)	20 (54)
Participant smokes any cigarette		
No	35 (95)	35 (96)
Yes	2 (5)	1 (2)
Former smoker	0	1 (2)

Abbreviations: NSA, nonspecific auricular acupuncture; SA, specific auricular acupuncture.

^a^ A score of 10 to 14 indicates moderate depression; 15 to 19, moderately severe depression; and 20 to 25, severe depression.

^b^ The monthly minimum wage in Brazil is equivalent to $250 in 2023.

### Primary Outcome

Three months after randomization, 14 of the 24 participants (58%) randomized to the SA group had a reduction of at least 50% in the PHQ-9 score compared with 10 of 23 participants (43%) in the NSA group. The result was not statistically significant (risk ratio [RR], 1.34; 95% CI, 0.76-2.45; *P* = .38) (Table 2).

**Table 2.** Primary and Secondary Efficacy Outcomes in the Intention-to-Treat Analysis

Outcomes	Participants, No./Total No. (%)		RR (95% CI)^a^	*P* value^b^
	SA group	NSA group		
Primary outcome, depression recovery at 3 mo vs baseline (n = 47)^c^^,^^d^	14/24 (58)	10/23 (43)	1.34 (0.76-2.45)	.38
Secondary outcomes				
Depression recovery at 4 wk vs baseline (n = 57)	12/28 (43)	9/29 (31)	1.28 (0.74-2.14)	.42
Depression recovery at 6 wk vs baseline (n = 51)	10/26 (38)	10/25 (40)	0.96 (0.53-1.65)	>.99
Symptom remission at 4 wk vs baseline (n = 57)^e^	8/28 (28)	5/29 (17)	1.35 (0.72-2.20)	.36
Symptom remission at 6 wk vs baseline (n = 51)	7/26 (27)	5/25 (20)	1.19 (0.61-2.00)	.74
Symptom remission at 3 mo vs baseline (n = 47)	11/24 (46)	3/23 (13)	1.99 (1.16-3.34)	.02

Abbreviations: NSA, nonspecific auricular acupuncture; RR, risk ratio; SA, specific auricular acupuncture.

^a^ Effect size was calculated as RR for dichotomous data.

^b^ Statistical significance was calculated with Fisher exact test.

^c^ The primary outcome (improvement in depression symptoms) was defined as a reduction of 50% or more from baseline Patient Health Questionnaire-9 (PHQ-9) scores. The number of cases and proportions are based on observed data.

^d^ Depressive symptoms measured by PHQ-9 scores range from 0 (least) to 27 (greatest) symptom burden.

^e^ Participants who achieved a score less than 5 on the PHQ-9, indicating symptom remission.

### Secondary Outcomes

**Figure 2.**
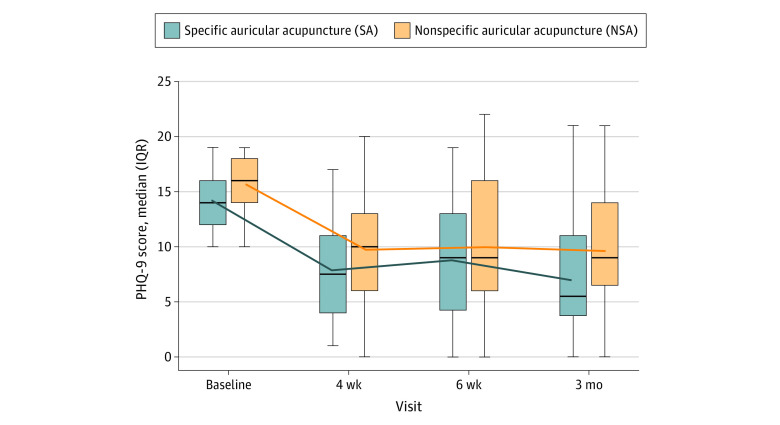
Median Depression Scores by Intention-to-Treat Analysis The figure shows the median Patient Health Questionnaire-9 (PHQ-9) scores of patients receiving specific auricular acupuncture (SA) and nonspecific auricular acupuncture (NSA) at baseline, 4 weeks, 6 weeks, and 3 months. A score of 10 to 14 indicates moderate depression; 15 to 19, moderately severe depression; and 20 to 25, severe depression. The lines inside the boxes reflect the medians, with the upper and lower ends of the boxes reflecting the IQRs. The error bars reflect the minimum and maximum values. The line plot reflects means.

The proportions of patients with depression recovery and remission based on the PHQ-9 at 4 weeks was higher in the SA group, but not at 6 weeks, and there were no statistically significant differences between the groups at 4 or 6 weeks. A statistically significant difference in favor of the SA group was observed in depression remission after 3 months (11 of 24 participants [46%] in the SA group vs 3 of 23 participants in the NSA group [13%]; RR, 1.99; 95% CI, 1.16-3.34; *P* = .02) (Table 2). The median PHQ-9 scores decreased after 4 weeks, 6 weeks, and 3 months in both groups, with no statistically significant differences. Lower scores were observed in the SA group than in the NSA group (eTable 6 in Supplement 2 and Figure 2).

The modified intent-to-treat analyses showed the similar results as the intention-to-treat analyses. Intragroup analysis was performed using the Cochran *Q* test, with no statistically significant differences (eTable 7 in Supplement 2). Intragroup analysis was performed using the Friedman analysis of variance test followed by post hoc tests for the differences in PHQ-9 scores at each time point compared with baseline, which revealed statistically significant differences in all evaluations, in both groups, at different assessment points (eTable 8 and eFigure 4 in Supplement 2).

### Sensitivity Analyses

The sensitivity analyses revealed that there were no statistically significant differences in depression recovery across the visits (eTable 9 in Supplement 2). These analyses confirmed the statistically significant results in favor of the SA group regarding depression remission after 3 months (eTable 10 in Supplement 2). As for PHQ-9 scores, the generalized estimating equations model demonstrated favorable effect sizes of SA, and the visits had a statistically significant effect on the scores (eTable 11 and 12 in Supplement 2).

### Safety

Most participants reported mild pain at the needle application site (33 patients [94%] in the SA group vs 32 patients [91%] in the NSA group). One participant had mild local inflammation in 1 of the auricular points and received appropriate treatment. Regarding adverse events, an increase in depression scores was observed during data collection in 3 participants, and 7 individuals responded positively to question 9 of the PHQ-9, which addresses suicidal ideation. These cases were carefully monitored, and the independent data monitoring and safety committee did not consider these events related to the intervention. No statistically significant difference was found between the groups in the proportion of participants with adverse effects (ie, those arising from AA) and adverse events (ie, worsening of depression symptoms or suicidal ideation). Five participants withdrew from the study as a result of adverse effects. No severe adverse events were reported (Table 3). The study participants did not change their medications or psychotropic medication dosages throughout the data collection.

**Table 3.** Adverse Effects and Adverse Events Listed by Treatment Group

Adverse event or adverse effect	Participants, No. (%) (N = 74)	
	SA group (n = 37)	NSA group (n = 37)
Mild and transient adverse effects		
Mild pain	33 (94)	32 (91)
Intense pain	1 (3)	0
Punctate bleeding	3 (9)	2 (6)
Itching in the auricle	11 (31)	14 (40)
Increased appetite	1 (3)	0
Headache	1 (3)	0
Hot flashes	0	1 (3)
Difficulty sleeping	0	2 (6)
Sensitivity to pressure	1 (3)	2 (6)
Facial skin hypersensitivity	0	1 (3)
Moderate adverse effect: local inflammation	1 (3)	0
Moderate adverse events		
Worsening of depressive symptoms (4 wk)	0	0
Worsening of depressive symptoms (6 wk)	0	1 (6)
Worsening of depressive symptoms (3 mo)	1 (3)	1 (6)
Emergence of suicidal ideation (4 wk)	0	2 (6)
Emergence of suicidal ideation (6 wk)	0	2 (6)
Emergence of suicidal ideation (3 mo)	2 (6)	2 (6)
Any severe adverse events and adverse effects	0	0
Discontinuation due to adverse effects		
Local pain	3 (9)	1 (3)
Local inflammation	1 (3)	0

Abbreviations: NSA, nonspecific auricular acupuncture; SA, specific auricular acupuncture.

### Blinding Quality

For results on the success of blinding, see eTable 13 in Supplement 2. No difference was found between groups in the proportion of patients who guessed correctly what kind of AA they had received and their perception of efficacy in symptom relief after the 3 months.

## Discussion

The results of this randomized clinical trial showed no difference in depression recovery between the groups at 3 months. However, depression remission at 3 months was significantly higher in the SA group. In addition, no significant differences were observed in the rates of adverse events and adverse effects, and no severe adverse events were recorded, evidencing practice safety. To our knowledge, this is the first randomized clinical trial on the effectiveness of SA using a semipermanent needle with NSA directed at depression as the comparator group. However, previous studies^22,23,24,25,26,27,28^ using other AA techniques found favorable results in depressive symptoms, but with shorter study duration and different comparators.

These preliminary findings are promising and support the use of SA in depression treatment. The rates of recovery (58%) and remission (46%) from depression in the present study are similar to those observed in meta-analyses of pharmacological treatments^29,30^ and superior to those for psychotherapy.^31^ The 33% difference in symptom remission between the groups (ie, 46% remission in the SA group vs 13% in the NSA group) was clinically meaningful; depression remission is crucial for patients, providing better psychosocial functioning and long-term prognosis, not just symptom improvement.^32^

The absence of statistically significant differences between the groups for depression recovery can be explained. First, the small sample size resulted in insufficient power and may have underestimated true efficacy. Second, considering the economic cost of time and participant adherence, we provided only 12 sessions over 6 weeks in this study. Therefore, adding AA doses by increasing the treatment duration (eg, 16-18 sessions over 8-12 weeks) may be an effective way to streamline the treatment program. Third, the manual stimulation of the semipermanent needle (known as de qi induction) is a factor that affects the efficacy of AA. To maintain the blinding of the study, the NSA group also used this daily stimulation, which may have contributed to an increased effect size of the technique in this group. Fourth, the study’s follow-up loss (36%) may have impacted the results. However, adherence to depression treatment remains a relevant and common challenge to overcome in clinical trials. A study by Olfson et al^33^ found that 36.4% of patients discontinued antidepressant treatment within the first 30 days. A meta-analysis^34^ reported a 36.4% dropout rate for cognitive behavioral therapy. A study^35^ on acupuncture in depression treatment observed a dropout rate similar to the present study. This trend of treatment discontinuation seems to be associated with the characteristics of the disease itself (apathy, hopelessness, and lethargy). Regardless of the chosen treatment, patients with depression are likely to discontinue treatment.^36^

First-line therapy for depression is medication, but several factors interfere with treatment access and adherence, such as system barriers and clinical and individual factors.^37^ Therefore, it is necessary to offer a wide range of evidence-based therapies to treat depression, aiming at person-centered mental health care.^38^ AA may be an option for patients who reject medication or psychotherapy because it has fewer adverse effects than pharmacological treatment. A systematic review^39^ of adverse events associated with AA found that most were transient, mildly intense, and tolerable. No severe adverse events were identified in our study, indicating that AA is a safe treatment.

Besides the technique’s safety, patients in the SA group had increased rates of symptom remission between 6 weeks and 3 months, which may be associated with the delayed effects of AA treatment.^40^ We should underscore that in AA, part of the technique’s effect is attributed to stimulating the auricular branch of the vagus nerve, increasing parasympathetic activity while simultaneously decreasing sympathetic nervous system activity.^41,42^ This effect may lead to an improvement in depression-related symptoms.^13^ A systematic review^43^ concluded that trigeminal nerve stimulation and transcutaneous vagus nerve stimulation improve the treatment of specific neuropsychiatric disorders, such as depression.

An additional hypothesis is that AA may reduce serotonin depletion by inhibiting the hyperactive hypothalamic-pituitary-adrenal axis, which would explain the technique’s prolonged effect at the 3-month follow-up.^44^ Experimental studies confirmed this hypothesis, showing that the antidepressant effect of AA is possibly associated with the normalization of this axis’ hyperactivity.^45^ Additionally, some studies^10,13^observed that AA can trigger cardioinhibitory effects similar to vagus nerve stimulation. It is essential to highlight that participants in the NSA group may have also experienced temporary inhibition of this axis due to the perception of having received adequate treatment. However, this effect tends to decrease gradually after the end of the treatment sessions.

This study has strengths. First, the research addressed the selection of AA points, providing a solid foundation for future large-scale studies. In addition, the study included a more extended study period and experienced acupuncturists who used semipermanent needles, which are considered more effective than other stimuli. The control group received needling in nonspecific areas, and blinding measures were implemented for assessors, participants, and the statistician.

### Limitations

The present study also has limitations. The predominance of women in the sample precluded the detection of possible gender differences in the results. Using self-report instruments to measure the primary outcome may also introduce bias into the results. Other major limitations include the limited sample size and the relatively high loss of participants during the follow-up period. Additionally, an NSA group with positive results indicates the need to investigate better the mechanisms of action and nonspecific outcomes associated with AA.

## Conclusions

The results of this randomized clinical trial suggest that SA over 6 weeks is feasible and safe. Although the differences between SA and NSA groups did not reach statistical significance for the primary outcome, SA did produce greater symptom remission at 3 months. A more extensive sample size study, more protracted intervention, and objective evaluation of the results are necessary to further investigate the efficacy of SA for depression.

## Notes

Supplement 1.
Study Protocol

Supplement 2. **eTable 1.** Inclusion and Exclusion Criteria **eFigure 1**. Data Collection Procedures **eFigure 2.** Points Applied in the Auricular Pavilion of the Specific Auricular Acupuncture (SA) Group **eTable 2.** Universal Nomenclature, Location, and Indications of the Points Used in the Groups **eFigure 3.** Points Applied in the Auricular Pavilion of the Nonspecific Auricular Acupuncture (NSA) Group **eTable 3.** Primary and Secondary Outcomes of the Study **eTable 4.** Loss to Follow-Up Throughout the Study **eTable 5.** Baseline Depression Score Analysis for Participants Who Did Not Complete the Study **eTable 6.** Secondary Outcomes of Depression Scores by Intention-to-Treat Analysis **eTable 7.** Secondary Efficacy Outcomes in Depression Recovery and Remission by Modified Intent-to-Treat Analysis **eTable 8.** Secondary Outcomes of Depression Scores by Modified Intent-to-Treat Analysis **eFigure 4.** Median Depression Scores by Modified Intent-to-Treat Analysis **eTable 9.** Sensitivity Analysis for Depression Recovery With Missing Data Imputation **eTable 10.** Sensitivity Analysis for Symptom Remission With Missing Data Imputation **eTable 11.** Sensitivity Analysis for Depression Score With Missing Data Imputation **eTable 12.** Adjustment of the Generalized Estimating Equations (GEE) Model **eTable 13.** Blinding Assessment and Perception of Treatment Efficacy

Supplement 3.
Data Sharing Statement

## References

[1] World Health Organization. Depression and other common mental disorders: global health estimates. 2017. Accessed October 30, 2023. https://apps.who.int/iris/handle/10665/254610

[2] GBD 2019 Mental Disorders Collaborators. Global, regional, and national burden of 12 mental disorders in 204 countries and territories, 1990-2019: a systematic analysis for the Global Burden of Disease Study 2019. Lancet Psychiatry. 2022;9(2):137-150. doi:10.1016/S2215-0366(21)00395-3 35026139 PMC8776563

[3] World Health Organization. World mental health report: Transforming mental health for all. June 16, 2022. Accessed July 30, 2023. https://www.who.int/publications/i/item/9789240049338

[4] World Health Organization. Mental health. Accessed July 30, 2023. https://www.who.int/health-topics/mental-health#tab=tab_1

[5] de Fátima Cunha M, de Cássia Gandini R. Compliance and non compliance to the pharmacological treatment for depression. Psic Teor e Pesq. 2009;25(3):409-418. doi:10.1590/S0102-37722009000300015

[6] Kessler RC, Soukup J, Davis RB, . The use of complementary and alternative therapies to treat anxiety and depression in the United States. Am J Psychiatry. 2001;158(2):289-294. doi:10.1176/appi.ajp.158.2.289 11156813

[7] Jorm AF, Korten AE, Jacomb PA, Christensen H, Rodgers B, Pollitt P. “Mental health literacy”: a survey of the public’s ability to recognise mental disorders and their beliefs about the effectiveness of treatment. Med J Aust. 1997;166(4):182-186. doi:10.5694/j.1326-5377.1997.tb140071.x 9066546

[8] Jorm AF, Medway J, Christensen H, Korten AE, Jacomb PA, Rodgers B. Public beliefs about the helpfulness of interventions for depression: effects on actions taken when experiencing anxiety and depression symptoms. Aust N Z J Psychiatry. 2000;34(4):619-626. doi:10.1080/j.1440-1614.2000.00761.x 10954393

[9] Yang Y, Wen J, Hong J. The effects of auricular therapy for cancer pain: a systematic review and meta-analysis. Evid Based Complement Alternat Med. 2020;2020:1618767. doi:10.1155/2020/1618767 32565846 PMC7267873

[10] La Marca R, Nedeljkovic M, Yuan L, Maercker A, Elhert U. Effects of auricular electrical stimulation on vagal activity in healthy men: evidence from a three-armed randomized trial. Clin Sci (Lond). 2010;118(8):537-546. doi:10.1042/CS20090264 19895369

[11] Peuker ET, Filler TJ. The nerve supply of the human auricle. Clin Anat. 2002;15(1):35-37. doi:10.1002/ca.1089 11835542

[12] Carreno FR, Frazer A. The allure of transcutaneous vagus nerve stimulation as a novel therapeutic modality. Biol Psychiatry. 2016;79(4):260-261. doi:10.1016/j.biopsych.2015.11.016 26796874

[13] Wu C, Liu P, Fu H, . Transcutaneous auricular vagus nerve stimulation in treating major depressive disorder: a systematic review and meta-analysis. Medicine (Baltimore). 2018;97(52):e13845. doi:10.1097/MD.0000000000013845 30593183 PMC6314717

[14] Corrêa HP, Moura CC, Azevedo C, Bernardes MFVG, Mata LRFPD, Chianca TCM. Effects of auriculotherapy on stress, anxiety and depression in adults and older adults: a systematic review. Rev Esc Enferm USP. 2020;54:e03626. 33111737 10.1590/S1980-220X2019006703626

[15] MacPherson H, Altman DG, Hammerschlag R, ; STRICTA Revision Group. Revised STandards for Reporting Interventions in Clinical Trials of Acupuncture (STRICTA): extending the CONSORT statement. PLoS Med. 2010;7(6):e1000261. doi:10.1371/journal.pmed.1000261 20543992 PMC2882429

[16] Kroenke K, Spitzer RL, Williams JB. The PHQ-9: validity of a brief depression severity measure. J Gen Intern Med. 2001;16(9):606-613. doi:10.1046/j.1525-1497.2001.016009606.x 11556941 PMC1495268

[17] Focks C. Atlas de Acupuntura: Com Sequência de Fotos e Ilustrações, Textos Didáticos e Indicações Clínicas. Manole; 2005.

[18] Lv X, Wang B, Jianbin B, Ye J. Clinical observation of depression after breast cancer operation treated with aurieular point sticking therapy. Article in Chinese. Zhongguo Zhen Jiu. 2015;35(5):447-450. 26255515

[19] Zhang CS, Yang AW, Zhang AL, May BH, Xue CC. Sham control methods used in ear-acupuncture/ear-acupressure randomized controlled trials: a systematic review. J Altern Complement Med. 2014;20(3):147-161. doi:10.1089/acm.2013.0238 24138333 PMC3948482

[20] do Prado JM. Aplicação da acupuntura auricular verdadeira e sham no tratamento do estresse. In: Enfermeiros. Escola de Enfermagem da Universidade de São Paulo; 2014.

[21] Dincer F, Linde K. Sham interventions in randomized clinical trials of acupuncture—a review. Complement Ther Med. 2003;11(4):235-242. doi:10.1016/S0965-2299(03)00124-9 15022656

[22] Valiani M, Mansourian M, Ashtari F. The effect of auriculotherapy on stress, anxiety, and depression in MS patients: a double blind randomized clinical control trial (parallel design). Acta Med Mediter. 2018;34(2):561-567. doi:10.19193/0393-6384_2018_2s_89

[23] Carter K, Olshan-Perlmutter M, Marx J, Martini JF, Cairns SB. NADA ear acupuncture: an adjunctive therapy to improve and maintain positive outcomes in substance abuse treatment. Behav Sci (Basel). 2017;7(2):37. doi:10.3390/bs7020037 28621706 PMC5485467

[24] Bergdahl L, Broman JE, Berman AH, Haglund K, von Knorring L, Markström A. Auricular acupuncture and cognitive behavioural therapy for insomnia: a randomised controlled study. Sleep Disord. 2016;2016:7057282. doi:10.1155/2016/7057282 27242930 PMC4876000

[25] Rodríguez-Mansilla J, González López-Arza MV, Varela-Donoso E, Montanero-Fernández J, González Sánchez B, Garrido-Ardila EM. The effects of ear acupressure, massage therapy and no therapy on symptoms of dementia: a randomized controlled trial. Clin Rehabil. 2015;29(7):683-693. doi:10.1177/0269215514554240 25322869

[26] Jiao Y, Han Y, Li X, . Comparison of body, auricular, and abdominal acupuncture treatments for insomnia differentiated as internal harassment of phlegm-heat syndrome: an orthogonal design. Evid Based Complement Alternat Med. 2015;2015:578972. doi:10.1155/2015/578972 26640498 PMC4657063

[27] Mohr P, Rodriguez M, Slavíčková A, Hanka J. The application of vagus nerve stimulation and deep brain stimulation in depression. Neuropsychobiology. 2011;64(3):170-181. doi:10.1159/000325225 21811087

[28] Wang XR, Song DD, Tao TQ, . Qi-regulating and blood circulation-promoting therapy improves health status of stable angina pectoris patients with depressive symptoms. Evid Based Complement Alternat Med. 2021;2021:7319417. doi:10.1155/2021/7319417 34567219 PMC8460386

[29] Trivedi MH, Rush AJ, Wisniewski SR, ; STAR*D Study Team. Evaluation of outcomes with citalopram for depression using measurement-based care in STAR*D: implications for clinical practice. Am J Psychiatry. 2006;163(1):28-40. doi:10.1176/appi.ajp.163.1.28 16390886

[30] Rush AJ, Trivedi MH, Wisniewski SR, . Acute and longer-term outcomes in depressed outpatients requiring one or several treatment steps: a STAR*D report. Am J Psychiatry. 2006;163(11):1905-1917. doi:10.1176/ajp.2006.163.11.1905 17074942

[31] Cuijpers P, Karyotaki E, Ciharova M, Miguel C, Noma H, Furukawa TA. The effects of psychotherapies for depression on response, remission, reliable change, and deterioration: a meta-analysis. Acta Psychiatr Scand. 2021;144(3):288-299. doi:10.1111/acps.13335 34107050 PMC8457213

[32] Rush AJ, Kraemer HC, Sackeim HA, ; ACNP Task Force. Report by the ACNP task force on response and remission in major depressive disorder. Neuropsychopharmacology. 2006;31(9):1841-1853. doi:10.1038/sj.npp.1301131 16794566

[33] Olfson M, Marcus SC, Tedeschi M, Wan GJ. Continuity of antidepressant treatment for adults with depression in the United States. Am J Psychiatry. 2006;163(1):101-108. doi:10.1176/appi.ajp.163.1.101 16390896

[34] Fernandez E, Salem D, Swift JK, Ramtahal N. Meta-analysis of dropout from cognitive behavioral therapy: magnitude, timing, and moderators. J Consult Clin Psychol. 2015;83(6):1108-1122. doi:10.1037/ccp0000044 26302248

[35] Mischoulon D, Brill CD, Ameral VE, Fava M, Yeung AS. A pilot study of acupuncture monotherapy in patients with major depressive disorder. J Affect Disord. 2012;141(2-3):469-473. doi:10.1016/j.jad.2012.03.023 22521855

[36] de Lorent L, Agorastos A, Yassouridis A, Kellner M, Muhtz C. Auricular acupuncture versus progressive muscle relaxation in patients with anxiety disorders or major depressive disorder: a prospective parallel group clinical trial. J Acupunct Meridian Stud. 2016;9(4):191-199. doi:10.1016/j.jams.2016.03.008 27555224

[37] American Psychiatric Association. American Psychiatric Association Practice Guidelines for the Treatment of Psychiatric Disorders: Compendium 2006. American Psychiatric Publishing; 2006.

[38] World Health Organization. WHO traditional medicine strategy 2014-2023. May 13, 2013. Accessed October 30, 2023. https://www.who.int/publications/i/item/9789241506096

[39] Tan JY, Molassiotis A, Wang T, Suen LKP. Adverse events of auricular therapy: a systematic review. Evid Based Complement Alternat Med. 2014;2014:506758. doi:10.1155/2014/506758 25435890 PMC4241563

[40] Liu J, Qin W, Guo Q, . Divergent neural processes specific to the acute and sustained phases of verum and SHAM acupuncture. J Magn Reson Imaging. 2011;33(1):33-40. doi:10.1002/jmri.22393 21182118

[41] Rong PJ, Zhao JJ, Li YQ, . Auricular acupuncture and biomedical research: a promising Sino-Austrian research cooperation. Chin J Integr Med. 2015;21(12):887-894. doi:10.1007/s11655-015-2090-9 26631173

[42] Fernández-Hernando D, Fernández-de-Las-Peñas C, Pareja-Grande JA, García-Esteo FJ, Mesa-Jiménez JA. Management of auricular transcutaneous neuromodulation and electro-acupuncture of the vagus nerve for chronic migraine: a systematic review. Front Neurosci. 2023;17:1151892. doi:10.3389/fnins.2023.1151892 37397439 PMC10309039

[43] Shiozawa P, Silva ME, Carvalho TC, Cordeiro Q, Brunoni AR, Fregni F. Transcutaneous vagus and trigeminal nerve stimulation for neuropsychiatric disorders: a systematic review. Arq Neuropsiquiatr. 2014;72(7):542-547. doi:10.1590/0004-282X20140061 25054988

[44] Le JJ, Yi T, Qi L, Li J, Shao L, Dong JC. Electroacupuncture regulate hypothalamic-pituitary-adrenal axis and enhance hippocampal serotonin system in a rat model of depression. Neurosci Lett. 2016;615:66-71. doi:10.1016/j.neulet.2016.01.004 26773866

[45] Liu RP, Fang JL, Rong PJ, . Effects of electroacupuncture at auricular concha region on the depressive status of unpredictable chronic mild stress rat models. Evid Based Complement Alternat Med. 2013;2013:789674. doi:10.1155/2013/789674 23431349 PMC3570937

