# Supplementary material for: Efficacy and Safety of Auricular Acupuncture for Depression: A Randomized Clinical Trial
Source: JAMA Netw Open. 2023 Nov 30;6(11):e2345138. doi: 10.1001/jamanetworkopen.2023.45138 (PMC10690462; doi:10.1001/jamanetworkopen.2023.45138)
Supplement: Supplement 1. — Study Protocol [file jamanetwopen-e2345138-s001.pdf]

Efficacy and Safety of Auricular Acupuncture for Depression: A Randomized Clinical Trial

Study Protocol
Final Version

Faculdade de Medicina, Universidade de São Paulo, São Paulo, Brazil
Department of Preventive Medicine

Av. Dr. Arnaldo, 455 · Cerqueira César
São Paulo · Brasil · 01246 903

www.fm.usp.br

Confidentiality Statement: This document is the intellectual property of the Investigators and contains strictly confidential information. Access is allowed only to sponsors, investigators, ethical committees, and regulatory bodies. No disclosure should occur without written authorization from the responsible investigators.

Study Contact and Organization

Daniel Maurício de Oliveira Rodrigues – MPH.
Department of Preventive Medicine, Faculdade de Medicina, Universidade de São Paulo, São Paulo, Brazil.
Department of Biological and Health Sciences, University of Southern Santa Catarina, Palhoça, Brazil.
Department of Health Sciences, Cruzeiro do Sul University, São Paulo, Brazil.
Email: danielmor7@gmail.com

Paulo Rossi Menezes – MD; Ph.D.
Department of Preventive Medicine, Faculdade de Medicina, Universidade de São Paulo, São Paulo, Brazil
Email: pmenezes@usp.br

Ana Elise Machado Ribeiro Silotto – BSc.
Department of Preventive Medicine, Faculdade de Medicina, Universidade de São Paulo, São Paulo, Brazil.
Department of Biological and Health Sciences, University of Southern Santa Catarina, Palhoça, Brazil.
Email: ana.silotto@usp.br

Artur Heps – BSc.
Department of Preventive Medicine, Faculdade de Medicina, Universidade de São Paulo, São Paulo, Brazil
Email: arturheps@usp.br

Nathália Martins Pereira Sanches – MD.
Institute of Psychiatry of Santa Catarina
Email: namartins@hotmail.com

Mariana Cabral Schveitzer – PhD.
Department of Preventive Medicine, Faculdade de Medicina, Universidade de São Paulo, São Paulo, Brazil
Email: marycabral101@gmail.com

Alexandre Faisal-Cury – MD; Ph.D.
Department of Preventive Medicine, Faculdade de Medicina, Universidade de São Paulo, São Paulo, Brazil
Email: alexandre.faisal@fm.usp.br; faisal@usp.br

Committee composition and collaborators

Name	Affiliation	Function
Alexandre Faisal Cury	Faculdade de Medicina, Universidade de São Paulo - Brazil	Chairperson - Steering committee
Ana Elise Machado Ribeiro Silotto	Faculdade de Medicina, Universidade de São Paulo – Brazil	Data coordination committee
Artur Heps	Faculdade de Medicina, Universidade de São Paulo – Brazil	Data coordination committee
Daniel Maurício de Oliveira Rodrigues	Faculdade de Medicina, Universidade de São Paulo – Brazil	Principal investigator for the trial
Gabriela Chaves Reis	University of Southern Santa Catarina, Palhoça – Brazil	Recruitment and outreach team
Beatriz Inez Dalsasso	University of Southern Santa Catarina, Palhoça – Brazil	Recruitment and outreach team
Joana Loudes Lawless	University of Southern Santa Catarina, Palhoça – Brazil	Recruitment and outreach team
Isabel Transferetti Pose	University of Southern Santa Catarina, Palhoça – Brazil	Recruitment and outreach team
Maria Eduarda Antunes	External contractor	Data collection
Mariana Cabral Schveitzer	Faculdade de Medicina, Universidade de São Paulo – Brazil	Data interpretation
Nathalia Martins Pereira Sanches	External contractor	Research Physician - Psychiatrist
Paulo Rossi Menezes	Faculdade de Medicina, Universidade de São Paulo - Brazil	Protocol Committee
Pedro Henrique de Mesquita Pacheco	External contractor	Statistician; Data Monitoring

Roberto Ferreira	External contractor	Data Manager; Data Monitoring

1. Background

The coronavirus disease (COVID-19) pandemic has affected individuals' mental health worldwide due to its rapid spread, high mortality, disruption of social relationships, high costs to healthcare systems, and devastating economic impact[1]. A systematic review of 43 studies on the consequences of the COVID-19 pandemic on mental health found increased levels of depression among infected individuals. Additionally, individuals with pre-existing psychiatric disorders reported deteriorated psychiatric symptoms. Studies focusing on healthcare professionals found an increase in depression/depressive symptoms, while research with the general public revealed lower psychological well-being and higher depression scores compared to before the COVID-19 epidemic[2].

The adverse, direct, and indirect effects of the COVID-19 pandemic on mental health are evident in the present and may extend into the future, especially for individuals already suffering from depression. Suicidal ideation may have possibly increased during and after the COVID-19 outbreak. However, whether this increase will have been short-term or long-term (or both) remains uncertain. Nevertheless, the mental health system must be prepared to treat depression and thereby prevent suicide [3]

Depression is one of the most significant public health problems in the world. It is characterized as a severe mental disorder with an episodic, recurrent, or persistent course over time, causing functional impairments in behavioral, social, family, educational, and occupational spheres. Several models have been created to explain the syndrome, which can be of psychogenic, organic, or combined nature[4].

According to the World Health Organization (WHO), depression is the leading cause of disability worldwide, with over 300 million people (4.4% of the global population) currently living with this symptom, representing an increase of more than 18% between 2005 and 2015. It is more common among women (5.1%) than men (3.6%). The prevalence also varies according to age, income, WHO macro-regions, and countries, among other variables. Globally, depressive disorders are classified as the primary factor for years lived with disability (7.8% of all years lost). In the worst cases, depression can lead to suicide, which causes about 800,000 deaths annually and is the second leading cause of death among adolescents and young adults (15-29 years). Despite high prevalence, less than 10% of those affected by the disease receive adequate treatment [5].

In the USA, 8.1% of adults had depression between 2013 and 2016, with nearly twice as prevalent among women (10.4%) compared to men (5.5%). About 80% of adults reported difficulties with social activities due to depression symptoms[6]. A study with American women found a prevalence of 4.8% for major depressive disorder and 4.3% for mild depression, with only 32.4% of women with major depressive disorder and 20.0% with mild depression using antidepressants[7]. In Brazil, the prevalence of depression is one of the highest in the world: 5.8% of the Brazilian population suffers from depression, and 10.3% of the years of life lost by Brazilians are due to this disease [5,8].

The DSM-V (Diagnostic and Statistical Manual of Mental Disorders)[9] identifies several categories of depressive disorders, with the difference between them lying in the relationship between the frequency and severity of symptoms. Major Depressive Disorder (MDD), for instance, is characterized by one or more depressive symptoms, lasting for two weeks with depressed mood or loss of interest, and at least four additional depression symptoms: significant weight loss or gain without dieting; insomnia or hypersomnia; psychomotor agitation or retardation; fatigue and loss of energy; feelings of worthlessness or excessive or inappropriate guilt; diminished ability to think or concentrate, or indecisiveness; recurrent thoughts of death (not just fear of dying); recurrent suicidal ideation without a specific plan, or a suicide attempt, or a specific plan for committing suicide [9].

Several theories elaborate on the etiology of depression. One of the main neurobiological hypotheses states that a decrease in the expression of brain-derived neurotrophic factor (BDNF) contributes to depression. This hypothesis is supported by consistent findings of low BDNF levels in the serum of depressed patients compared to levels detected in non-depressed patients. Meta-analyses have confirmed abnormally low brain-derived neurotrophic factor (BDNF) concentrations in depressed patients and its normalization with antidepressant treatment. These findings appear to reflect peripheral manifestations, aligned with the neurotrophic hypothesis: depression results from altered BDNF expression in the brain[10,11].

In the pathophysiology of depression, besides BDNF, it has been increasingly confirmed in recent years that the inflammatory immune system, especially the release of signaling molecules (cytokines),

could influence many of the neurochemical changes induced by stress and contribute to developing depression, interacting with the neuroendocrine system and specific pathways related to mood[12]. Recent studies have argued that changes in the levels of pro-inflammatory cytokines (Interleukin 1β, Interleukin-6, and TNF-α) are associated with an increased risk of depression, both in animal models and in patients with depression[13,14].

Different types of depression are treated with specific medications called psychopharmaceuticals. Their use is related to the already established alteration of neurochemical neurotransmitters. With this support, scholars explain the nature of the disease and the action of antidepressants[15]. Medications are prioritized in treating major depressive disorders, which explains the current wide variety of these products [16].

Non-adherence to drug treatment has been included in the concerns of healthcare professionals, along with other factors that influence the rational use of therapeutic resources[17]. Adherence to antidepressant treatment is relatively low, ranging from 40 to 90% in different studies, with a mean of 65%[18].

Other biological treatments affect brain chemistry, such as electroconvulsive therapy (ECT) and vagus nerve stimulation (VNS). However, these interventions attach risks of developing infections and other side effects resulting from the surgical process[19,20].

Therefore, it is necessary to explore safe and effective methods for treating depression. Transcutaneous vagus nerve stimulation was inspired by Auricular Acupuncture from Traditional Chinese Medicine. It is a non-invasive method with efficacy in treating neuropsychiatric disorders[21]. Transcutaneous electrical stimulation of the auricular concha or the lower half of the posterior auricle (distribution of the afferent vagus nerve) can produce a similar modulating effect to invasive nerve stimulation [22]. A meta-analysis evidenced that transcutaneous auricular vagus nerve stimulation therapy could effectively improve symptoms of major depressive disorders, providing a technique for treating depression [23].

Some healthcare professionals indicate complementary biological and psychotherapeutic interventions as additional treatments to psychopharmaceuticals, which can encompass different methods such as psychotherapy, psychodynamic therapy, interpersonal therapy, behavioral therapy, cognitive-behavioral therapy, group therapy, couple therapy, family therapy, and interventions in life quality changes[24], creating new habits such as diet, exercise practices, and treatments from other Eastern or Western therapeutic systems known as integrative and complementary practices in Brazil.

The last three decades have witnessed significant discussion among academic, political, and technical groups related to the health sector about including other complex non-biomedical healthcare models, referred to by the WHO as Traditional Medicine (TM) and Integrative and Complementary Medicine (ICM). These complex systems are widely used in both developed and developing countries, in public and private settings. As such, the WHO established that member countries implement treatments consisting of TM and ICM not belonging to the biomedical model in their healthcare systems [25,26].

Since 2018, 170 WHO member states have recognized the use of TM/ICM, and 97 countries have a national policy related to the subject[27]. Studies show that TM/ICM use prevalence ranges from 9.8% to 76% worldwide[28]. Data from 32 countries found an overall prevalence of TCM use of 26.4% in the past 12 months, ranging from less than 10% in Poland and Slovenia to over 50% in China and the Republic of Korea[29]. In Brazil, the prevalence of using any TM/ICM was 5.3% in the past 12 months. Medicinal and herbal plants (57.2%), acupuncture (26.3%), and homeopathy (17.0%) were the most used among TCM users.

The data show that 17,350 services in the Health Care Network (RAS) offered some Integrative and Complementary Medicine (ICM) in 2019. This offer is available in 4,297 (77%) Brazilian municipalities, covering all twenty-seven states, the Federal District, and all capitals. In the service network, ICMs are predominantly present in Primary Health Care (PHC) (90%). A total 15,603 (37%) of the 41,952 Basic Health Units (UBS) operating in the SUS offer ICM. Auricular acupuncture was the most performed practice among the procedures performed in 2019 in ICM in PHC and Medium- and High-Complexity (MAC), with 915,779 procedures. From 2017 to 2019, the growth of this technique in PHC was over 930%, up from 40,818 to 423,774 out of a total of 628,239 attendances. Regarding ICM in MAC, 492,005 out of 1,463,183 attendances were related to auricular acupuncture[30].

The use of ICM has increased significantly worldwide in recent decades, whose demand has gained prominence in the field of health due to its differentiated approach: to understand the individual with a multidimensional, expanded, and singular approach to the life-health-disease process, to improve the quality of life and well-being and promote the recovery and preservation of people's health[31–33]. Other aspects encourage the search for such practices: a receptive approach, establishing a therapeutic bond, individual shared responsibility for health, awakening autonomy, and empowerment[32].

Besides the mentioned aspects, several studies show their efficacy, effectiveness, safety, and action mechanisms[26]. The Cochrane, an independent international nonprofit organization, has had an ICM

reference center at the University of Maryland since 1996, significantly contributing to decision-making by producing the best evidence through systematic reviews and meta-analyses, where among the most used and researched practices are those of Traditional Chinese Medicine [34].

Traditional Chinese Medicine (TCM) is a system of therapeutic practices used in China for thousands of years. It is considered one of the oldest traditional medicines and has gained increasing popularity in the West. Several theories underpin its practice. One is the yin/yang theory, which describes the complementary interaction between opposing and complementary forces in the universe and the human body. It seeks a balance between these forces to promote health and well-being.

Additionally, TCM uses the five-element theory, which associates different body organs and systems with elements of nature, such as Wood, Fire, Earth, Metal, and Water. Another central aspect of TCM is the Zang Fu theory, which describes the organs and viscera as interconnected systems, each with specific functions. It considers the physical organs, their mental and emotional functions, and their relationships with the environment and society. These organs and viscera favor the correct circulation of Chi (vital energy). Chi is believed to flow through the body meridians, forming a network that connects all organs and systems. The balance and free circulation of Chi in the meridians of the organs and viscera are essential for health and well-being [35].

The Lung is one of the fundamental organs in TCM. It is responsible for the exchange of Chi between the body and the external environment. Besides its respiratory function, the Lung controls the Chi of the entire body and the Defense Chi, protecting the body against external invasions. It is also related to sadness, melancholy, feelings of worthlessness, sorrow, and excessive focus on the past[36].

The Heart is considered the monarch of organs and governs the circulatory system and the mind. It pumps blood and houses the Mind, which controls consciousness, sleep, and emotions. The Heart is responsible for joy, the expression of emotions, and sleep quality. TCM affirms that a healthy Heart is essential for a balanced mind[35,37].

The Kidneys play a fundamental role in storing Jing (ancestral essence) and regulating bodily fluids. Additionally, they are responsible for the production and circulation of Jing, which provides vital energy to the whole body. They are associated with vitality, healthy aging, and hormonal balance, but also to fear, insecurity, willpower, or loss of interest[38,39].

Finally, the Liver regulates the free flow of Chi and Blood, promoting balance and harmony in the body. Additionally, the Liver stores Blood and is related to the free circulation of emotions. A healthy Liver is fundamental for good mental health and the health of tendons and eyes. This organ is related to irritability, mood instability, frustration, and resentment [40].

Each organ is seen as a complex system with multiple functions, interconnected and interdependent with other organs and systems of the body. In TCM, health is achieved with balance and harmony between the organs, and disease is seen as an energetic imbalance. The TCM diagnosis considers the specific functions of each organ and seeks to restore Chi balance to promote the individual’s health and well-being [35].

This diagnosis includes observing (face, eyes, tongue, and auricular pavilion), listening (voice tone), smelling, questioning the individual’s history, and palpating the pulse, chest, and abdomen. Based on the collected information, a diagnosis is elaborated using the classification of signs and symptoms based on the theoretical principles mentioned above (Yin/Yang, five elements, Zang Fu, and meridians)[41].

Regarding the diagnosis of depression, in the TCM approach, depression is caused by an imbalance in the flow of Chi and the functioning of body organs and systems. TCM affirms that several syndromes are involved in depression. They include the Kidney Chi deficiency syndrome, which may be associated with fatigue, weakness, lack of motivation, and low self-esteem; the Liver Chi stagnation syndrome, which can cause irritability, restless sleep, and mood instability; the Heart Chi and Blood deficiency syndrome, which may manifest as profound sadness, mental agitation, insomnia, and discouragement; Lung Chi deficiency, which can lead to lack of vitality and enthusiasm, resulting in symptoms such as sadness, feelings of worthlessness, melancholy, and lack of motivation; finally, Lung Chi stagnation, causing emotional symptoms such as anguish, sadness, and a feeling of oppression in the chest[42].

Treatment is grounded on the diagnosis, meaning that the theories mentioned above also influence the choice of therapies. The primary therapies of the TCM treatment system mainly include the use of herbal medicine (Chinese Herbal Medicine), diet, mind-body practices (meditation, Qi Gong, Tai Chi Chuan, Lian Gong, Chi Kung), massage techniques, acupuncture, moxibustion (heat application), cupping (suction technique), Gua Sha (scraping technique); auriculotherapy or auricular acupuncture [43].

Auricular acupuncture is an ancient therapy. It is the art of balancing the body through reflex points distributed in the auricular pavilion[44]. The National Cancer Institute – NIH (2021)[45] defines it as a type of acupuncture in which fine needles are inserted into specific points on the auricular pavilion to control pain and other symptoms. Like reflexology, the human body and all its organs and limbs are believed to be

projected onto the auricular pavilion, and each region corresponds to a specific point. Furthermore, stimulation in specific points can produce systemic effects or effects in corresponding reflex regions[46].

Several nerves, including spinal and cranial, are distributed on the auricular pavilions, particularly in the triangular fossa and concha region. The vagus nerve is a mixed nerve composed of about 80% afferent fibers among these nerves. The concha area has a rich distribution of the vagus nerve[47]. Anatomical studies have shown that the auricular pavilion is the only place on the surface of the human body with a distribution of the afferent vagus nerve[48]. Thus, stimulation by a needle on the auricular pavilion seems to lead to specific activation in the brain, mainly through the auricular branch of the vagus nerve[22,23], producing a similar effect to classic vagus nerve stimulation in reducing depressive symptoms without the burden of surgical intervention[21].

As mentioned earlier, auricular acupuncture action mechanisms are similar to transcutaneous vagus nerve stimulation. Both are non-invasive techniques applied to the auricular pavilion. Auricular acupuncture typically uses fine needles or other stimuli on the surface of the auricular pavilion, such as seeds, metal beads, crystals, lasers, and electrostimulation[49]. Transcutaneous vagus nerve stimulation uses electrical stimulation applied through electrodes on the cymba conchae and cavum conchae regions of the auricular pavilion [50].

Kraus et al. (2007)[51] found that transcutaneous vagus nerve stimulation can induce reductions in functional magnetic resonance imaging signal in limbic areas of the brain, including the amygdala, hippocampus, parahippocampal gyrus, and middle and superior temporal gyri, and increased functional magnetic resonance imaging signal in the insula, prefrontal gyrus, and thalamus. All these structures are essential for treating depression. Fang et al. (2016)[52] suggested that the modulation caused by transcutaneous vagus nerve stimulation influenced the networks of brain regions associated with emotional regulation/affect.

Auricular acupuncture differs from systemic body acupuncture in several aspects. In auricular acupuncture, semi-permanent needles are retained for extended periods and can remain in the ear for hours or even days after the treatment session. In contrast, the needles are inserted and removed during the session in body acupuncture. Additionally, body acupuncture requires years of education and specialized training to become a qualified professional, whereas auricular acupuncture is a more straightforward technique, often taught in a few days[53].

Studies suggest that the long-term incorporation of semi-permanent needles into the subcutaneous myofascial layer in the auricular pavilion can maximize the therapeutic effects of auricular acupuncture. Continuous stimulation over time can regulate neurochemical functions, promote blood circulation, and achieve a better healing effect[54]. Moreover, a clinical trial conducted by Kurebayashi et al. (2014)[55] compared the effects of auricular acupuncture with semi-permanent needles and auricular acupuncture with seeds. The study found that the use of needles showed better effects on mental health compared to the use of seeds.

Research in the databases PubMed, Medline, LILACS, Ibecs, Cumed, SciELO, and Cochrane found 32 articles with the keywords in English, Spanish, and Portuguese: "*ear acupuncture*" or "*auricular acupuncture*" or "*auriculotherapy*" and "*depressi**". Chinese scientific articles prevail, with heterogeneous data collection. A systematic review found weak evidence of the efficacy of auricular acupuncture for depression. The methodological quality scores showed that most of the seven studies analyzed indicated considerable methodological flaws. Most of the cited studies positively impacted depression scores. However, these findings were compromised by study design flaws, including a short treatment period, lack of blinding, lack of description of the randomization process, allocation concealment, and variable skills of acupuncturists[49].

Three other clinical trials of auricular acupuncture for depression were found besides the studies mentioned in the systematic review. The target population of the studies included 90 women with breast cancer, 90 male addicts, and 24 obese women. Auricular acupuncture had significant results in depression scores in all three studies, but the studies had a low number of participants and a high risk of biases [56–58].

Fu et al. (2009)[59] conducted a multicenter randomized clinical trial combining body acupuncture and auricular acupuncture with 440 individuals, divided into 176 people in the body and auricular acupuncture group, performed twice a week for three months; 176 people treated with Fluoxetine Hydrochloride, and 88 people in the control group (sham body and auricular acupuncture). The results showed that the therapeutic effects are better or similar to Fluoxetine Hydrochloride but with fewer side effects.

Other studies reported the effects of auricular acupuncture in different mental health situations and outcomes: improved preoperative anxiety, lower burnout and stress levels in healthcare professionals[60], improved sleep quality in individuals with post-traumatic stress disorder[61]; improved stress levels in nurses[55]; improved stress, cortisol levels, and sleep quality in middle-aged women[62].

Auricular acupuncture is currently one of the most widespread Complementary and Integrative Health Practices (CIPs) because it allegedly has quick and effective results, with rare reports of adverse reactions and contraindications. Research has shown that Australians and North Americans report a preference for CIPs to treat depression[63–65].

It is also worth noting that auricular acupuncture is easier to implement in clinical settings than body acupuncture due to its short application time, low technique complexity, relative safety, and continuous daily physiological stimulation[66]. Another considered advantage of auricular acupuncture is its application to psychiatric patients. The patient does not need to undress and can remain seated, similar to psychotherapy, i.e., the individual remains at eye level with the interlocutor without being in a lower and passive position, having to lie down without clothes[67].

Despite safety reports with the practice, acupuncture and auricular acupuncture practices are not entirely adverse event-free. White (2001)[68] reported 684 adverse events per 10,000 treatment sessions. Most were minor events, such as bleeding, needle pain, or symptom exacerbation. Another prospective study by MacPherson et al. (2001)[69] showed that no severe adverse events were reported after 34,407 acupuncture treatment sessions. This is significant evidence for public health, as acupuncture practitioners perform approximately two million treatments annually in the UK. Comparing this rate of adverse events with acupuncture to the rate of events from routinely prescribed medications in primary care, acupuncture is considered a relatively safe form of treatment.

Several mechanisms may explain the therapeutic effects of acupuncture technique in depression. In the last thirty years, substantial evidence has shown that the technique acts on various neurotransmitters, predominantly the endogenous opioid mechanism, catecholamines, serotonin, norepinephrine, and about 20 to 30 other neuropeptides[70]. Acupuncture has also produced structural and functional changes shown in magnetic resonance imaging and electroencephalography in areas of the anterior cingulate cortex, amygdala, hippocampus, hypothalamus, cerebellum, and other limbic structures[71,72]. Dysfunctions in these areas of the brain have been related to depressive disorders[73].

Despite the studies published on the technique's action mechanism, most studies on the efficacy and safety of different acupuncture modalities for depression show high risk of bias. A systematic review by Cochrane on this topic[74] concluded that most of the 64 studies had biases due to the low methodological quality of the published clinical trials. Most articles included in this review used other acupuncture modalities (manual acupuncture, electroacupuncture, and laser acupuncture) or combined with auricular acupuncture. Only one study using auricular acupuncture alone was analyzed in this review[56].

As a result, based on the clinical experience of professionals in the field and the positive acceptance by patients, auricular acupuncture has become a widely used practice due to its economic accessibility and ease of application. However, there are few clinical studies with this technique, and most published studies have many biases that compromise the interpretation of the results. In many studies, depression was evaluated as a secondary outcome of different health conditions. Finally, different techniques were employed, with only two studies using semi-permanent needles and without a control group. Therefore, the study is justified by the novelty of the proposal and the study design rigor, a randomized clinical trial with blinding of the assessor, statistician, and participant. This study aims to estimate the efficacy and evaluate the safety of auricular acupuncture for depression.

2. Data Collection Instruments

2.1. Assessment of Depression Levels

The Patient Health Questionnaire-PHQ-9[75] will be used to assess depression levels. It is a self-assessment instrument used worldwide. It is a brief, validated instrument widely used in clinical research and sensitive to changes over time[76,77].

The PHQ-9 is a nine-question questionnaire aimed at evaluating major depression typical symptoms, as established by the DSM-IV [78]. Each question seeks to identify the frequency of each symptom over the past two weeks, using a Likert scale with 0 to 3 points. The responses correspond to the following frequency levels: "not at all", "several days", "more than half the days", and "nearly every day", respectively. Besides the nine questions, the questionnaire also includes a tenth question that assesses how these symptoms interfere with daily activities, such as work and study. In Brazil, the instrument has been validated for use in adults [75].

The scores range from 0 to 27 points, classifying according to the levels of depression diagnosis: no depression (0-4), mild symptoms (5-9), moderate/moderately severe symptoms (10-19), and severe symptoms (20-27)[76,78].

The response to treatment, also called recovery, indicates a significant reduction in depression symptoms in response to therapeutic intervention. It is generally defined as a decrease of a specific

percentage in the score of a depression assessment scale, such as the PHQ-9. Recovery from depression can be defined as a reduction of 50% or more in the scale's total score[79].

Depression remission, on the other hand, represents a significant lack of depressive symptoms after treatment. It is usually defined as reaching a score below a specific threshold on a depression assessment scale, indicating no symptoms or only mild symptoms. A PHQ-9 score below 5 can be considered remission[79].

2.2. Assessment of Adverse Events and Effects

The adverse event assessment will evaluate the possible deterioration of depressive symptoms, measured by the PHQ-9; the appearance of suicidal ideation or self-harm, as evaluated by question 9 of the PHQ-9.

Interviewers may identify severely depressed or suicidal individuals during data collection, and specific actions to address these situations have been included in the protocol. The PHQ-9 scores obtained will be analyzed by the research supervisor immediately after the assessors collect the information. If any participant presents a risk during the study, an appointment will be scheduled with a psychiatrist researcher from the study to treat these symptoms. Additionally, the participant will be referred to the University's Psychology and Naturology clinic for treatment.

A questionnaire developed specifically for this study will be used in all sessions to assess the adverse effects of auricular acupuncture. Regardless of whether undesirable experiences occur during the study or not, they will all be recorded as adverse events or effects. Severe adverse events can be defined as situations involving (1) death, (2) life-threatening events, (3) hospitalization, (4) disability or permanent damage, (5) congenital anomalies/congenital disabilities, or (6) the need for medical care or surgical intervention. Moderate events will be considered when participants require intervention from the researchers.

A suspected causal relationship between the intervention and the adverse event or effect will be classified as intervention-related. Criteria such as the specific adverse effect having been previously reported in association with the intervention, a clear temporal relationship with the intervention, repeated occurrence in the intervention group, or a physiologically plausible causal relationship will be considered to consider an intervention-related adverse effect. This assessment will also consider the evaluation from the participant's perspective. If participants withdraw from the study due to adverse effects, this information will also be analyzed.

3. Study hypotheses

Auricular acupuncture is more effective than usual care and non-specific auricular acupuncture in reducing and remitting depressive symptoms, both in the short term (four and six weeks after inclusion) and in the medium term (three months after inclusion). It is also safe for treating patients with depression.

4. Study objectives

The primary outcome will be at least a 50% reduction in the PHQ-9 score (depression recovery) at 3 months.

4.1. Secondary Objectives

Secondary outcomes will include recovery (4 and 6 weeks) and remission (4 and 6 weeks and 3 months), adverse events, assessment of blinding, and perception of efficacy.

5. Declaration of interests

The researchers involved in this randomized clinical trial have no conflicts of interest that could influence the conduct or results of this study. No relationships could compromise the integrity and impartiality of the research. All team members responsible for the trial are committed to transparency and honesty in conducting the study, ensuring the protection of participants' interests and the quality of the obtained results.

6. Methodology

6.1. Study design

The present research is classified as a quantitative study according to its nature; experimental according to the procedures; and explanatory according to the objectives. This study is characterized as a randomized clinical trial, blinded to the participants, the evaluators, and the statistical analysts.

The project titled "Efficacy and Safety of Auricular Acupuncture in Depression: A Randomized Pilot", coordinated by Prof. Dr. Alexandre Faisal Cury, was approved by the CEP-HCFMUSP under N° 6.083.343 and by the CEP-UNISUL under N° 3.781.279. This research received funding from FAPESP under grant N° 2018/8117469-5. The FAPESP provides funding for this study and plays a fundamental role in its realization. All decisions related to the study design, data collection, analysis, and results dissemination will be made independently by the responsible researchers, ensuring the integrity and impartiality of the study. The research protocol was registered on the ClinicalTrials.gov platform under N° NCT05855421.

6.2. Study setting

University research centers in Santa Catarina, Brazil.

6.3. Recruitment

The research participants will be adults aged 18 to 50 years. The study will be advertised through posters on university bulletin boards, on its own website, on social media, and through brochures distributed in the university area. The sample will consist of individuals meeting the minimum scores and fitting the study's inclusion and exclusion criteria.

6.4. Retention

Strategies will be adopted to keep continuous contact with the research participants, favoring their retention throughout the study. Regular communication will be provided through phone calls, text messages, and emails to remind participants about upcoming sessions and assessments.

6.5. Randomization and blinding

For block randomization, a computer program http://www.randomization.com will perform randomization at a 1:1 ratio, with blocks of different sizes (4, 6, and 8), ensuring allocation concealment and a balanced number of participants in each group. The study will have two groups: the experimental group (auricular acupuncture – specific points for depression and usual care) and the control group (auricular acupuncture – non-specific points and usual care).

Participant allocation will be concealed, meaning it will be performed through a random numerical sequence generated by the computer program by an independent statistician. The allocation information will be placed in individual opaque and sealed envelopes, each containing a letter indicating the group to which each participant will be assigned. Neither the study participants nor the investigators will have any influence on the randomization and allocation concealment.

The participants will be blinded to the auricular acupuncture group (specific and non-specific points for depression). They will be informed that there are two treatment protocols.

The study evaluators will be blinded to the group assignments. The auricular acupuncture practitioners will need to find out the results of the participants' questionnaire scores. Lastly, the statistician will also be blinded to the participants' origin when preparing the results reports.

6.6. Eligibility Criteria

The study eligibility will be assessed through pre-screening in a dedicated room, preserving the participant's integrity, confidentiality, and anonymity. The following inclusion and exclusion criteria will be used for the study.

6.7. Inclusion Criteria

- Age between 18 and 50 years;
- Obtaining minimum scores on the PHQ-9 for moderate depression;

- Availability for the treatment sessions.

6.8. Exclusion Criteria

- Use of complementary therapies for a minimum of 3 months;
- Risk of suicidal ideation assessed through question 9 of the PHQ-9;
- Severe depression evaluated by the PHQ-9 scores;
- Prior application of auricular acupuncture;
- Pregnant individuals;
- Menopausal individuals;
- Allergy to metals and micropores;
- Healthcare students with a history of complementary therapies in their curriculum;
- Workers with scheduled vacations during the data collection period;
- Lack of access to the earlobe of the auricular pavilions due to mutilation, cartilage deformation, piercing from earring use, or other artifacts that would prevent the application of auricular acupuncture at the specific point.

7. Interventions

Volunteer eligible participants will be instructed about the research objectives and the respective ethical implications through the Informed Consent Form (ICF). After that, a questionnaire with demographic, socioeconomic, health, and behavioral variables will be administered to determine who meets the inclusion and exclusion criteria. The PHQ-9 will be used to identify participants with symptoms of depression. Researchers using the REDCap platform will apply all these instruments on tablets.

The study assessors have been trained to approach participants similarly to avoid recruitment biases. The study will have a protocol of questions used in all phases: pre-screening, screening, data collection, and application of protocols.

Participants with severe depression (PHQ≥20) or suicidal ideation will not be eligible to participate in the study and will be referred to and treated by a psychiatrist researcher of the study.

The acupuncture auricular sessions will take place twice a week, lasting 15 minutes each, in a dedicated room for the research, ensuring privacy and confidentiality, per the availability of participants and researchers, for 6 weeks.

The experimental group consisted of a protocol of points chosen according to the diagnosis of depression by TCM. In all participants, six pre-established points were used: Shenmen, Kidney, Liver, Heart, Lung, and Subcortex[56,58,80] (Figure 1). The EL11 device from the NKL brand was used for the exact location of the points, which searches for points with lower electrical resistance on the skin, indicating more neuroreactive points, that is, true acupuncture points[81]. The size of the semi-permanent needle used was (0.20mm X 2.5mm) from the Complementar brand. The semi-permanent needles were inserted to a depth of 2.5mm (Table 1).

Figure 1. Points applied on the auricular pavilion in the specific acupuncture group.

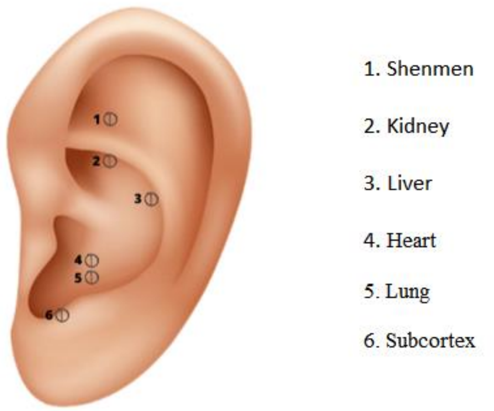

Table 1. Universal nomenclature, location, and indications of the points used in the groups.

Group	Ear Acupuncture Point	Universal and Chinese Nomenclature	Anatomical Location	Indications according to TCM and scientific studies	Indications for mental health and depression
**Specific AA**	*Shenmen*	*TF4 Shenmen*	The upper portion of the apex of the triangular fossa is closer to the upper branch of the antihelix.	Analgesic and anti-inflammatory action is the most critical anesthesia point on the ear.	Sedative point. Calms the mind.
	Subcortex	*AT4 Pizhixia*	Inner and anterior faces of the antitragus	Rebalances nervous, digestive, and cardiovascular functions	Harmonizes cortical excitation and inhibition
	Heart	*CO15 Xin*	In the center of the cavum conchae	Used for cardiac complaints such as angina, cardiac rhythm disorders	Indicated for anxiety, depression, distress, mental agitation, and insomnia.
	Lung	*CO14 Fei*	Above and below the heart point	Assists with respiratory tract diseases, indicated for nicotine dependence and skin conditions	Indicated for melancholy, sadness, grief, attachment to the past
	Liver	*CO12 Gan*	In the cymba conchae, above the root of the helix, close to the antihelix.	Used for liver-related issues, intercostal neuralgia, and ocular conditions.	Indicated for irritability, frustration, resentment, impatience, and mood swings.
	Kidney	*CO10 Shen*	In the cymba conchae, in a groove below the start of the inferior crus.	Used for issues in the urinary and genital systems. Assists with tinnitus, auditory conditions, and sleep disorders.	Indicated for fear, insecurity, and lack of willpower.
**Non-specific AA**	External Ear	*LO6 Neier*	In the depression between the tragus's upper end and the helix's ascending spine.	Used for inflammatory changes in the external ear, it assists with tinnitus, hearing loss, and temporomandibular joint pain.	No known indication for depression and mental health.
	Cheek/Face	*LO5 e LO6 Mianjia*	In an oval portion in the region of the 2nd, 3rd, 5th, and 6th quadrants.	Used for myofascial spasms, facial paresis, and trigeminal neuralgia.	No known indication for depression and mental health.
	4 Non-specific points in the Helix region	*HX9 Lunyi HX10Lunar HX11 Lunsan HX12 Lunsi*	On the margin of the auricle, between the helix tubercle and the apex, and between the tubercle and the lower lobe point.	Used for thirst control.	No known indication for depression and mental health.

International literature acknowledges the difficulty in establishing protocols to be used as controls due to the high responsivity and innervation of the auricular pavilion; therefore, sham needling is not physiologically inert[82]. Regarding the control group in auricular acupuncture, sham interventions typically include non-penetrating sham needles, superficial needling, needling in non-acupoints, sham interventions without needling, minimal acupuncture (without puncturing), and needling in irrelevant true acupoints[58,83].

Thus, for the control group in the study, the strategy chosen was superficial needling in "non-acupoints" or "irrelevant true acupoints", aiming to target other neural segments. The control group points (auricular acupuncture – non-specific points) were selected based on a previous protocol. The points included the external ear and cheek/face area[82] and four non-specific points in the helix region[84] (Figure 2), which are points not directly related to mental health symptoms (Table 1). The locator device confirmed that the sham areas were not neuroreactive points. The needle size for the control group was (0.20mm X 1.0mm) from the Complementar brand, aiming for more superficial needling. The semi-permanent needles were inserted to a depth of 1.0mm.

Figure 2. Points applied on the auricular pavilion of the non-specific acupuncture group.

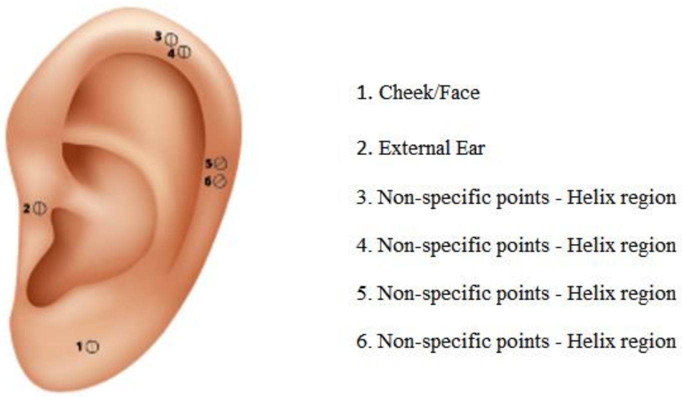

The first application will be performed on the participants' right ear; in the following sessions, the ears will be alternated with each application. Each participant will be instructed to keep the needles in place, emphasizing the need for pressure stimulation in the points' region. The research applicators will remove the needles from the previous session and apply them to the same points on the other ear, ensuring constant stimulation to the free nerve endings. The research applicators will instruct the participants to manually stimulate each point for 30 seconds or until the ear becomes red or sensitive to pressure three times a day (morning, afternoon, and evening) every day. This stimulation is known as "Deqi".

The Deqi stimulus is a specific sensation experienced by patients during acupuncture application. In Traditional Chinese Medicine (TCM), Deqi is believed to be an essential component in achieving the desired therapeutic effects. The Deqi sensation is described as a combination of sensations, including pain, tingling, heaviness, pressure, warmth, or coldness, occurring at the site where the needles are inserted. Proper Deqi stimulation is associated with the clinical efficacy of acupuncture as it indicates a physiological response of the body[85].

All instructions will be provided before opening the participant allocation envelope, preventing acupuncturists from differentiating procedures based on the group. Participants will be advised to refrain from engaging in direct conversations or seeking clarification from the acupuncturists. However, they should approach the research assistant, who will be available in person at the research site or through WhatsApp or phone.

All participants will respond to the PHQ-9 questionnaires at four and six weeks and three months after the start of the study, enabling analysis and comparisons of symptom progression and possible adverse effects (increased depression and risk of suicidal ideation). The assessors will administer the questionnaire before each auricular acupuncture session to identify any potential undesired events related to the technique (Figure 3). All participants will continue with their usual care for ethical reasons for the evaluation of adverse events.

Figure 3. Flowchart of data collection procedures

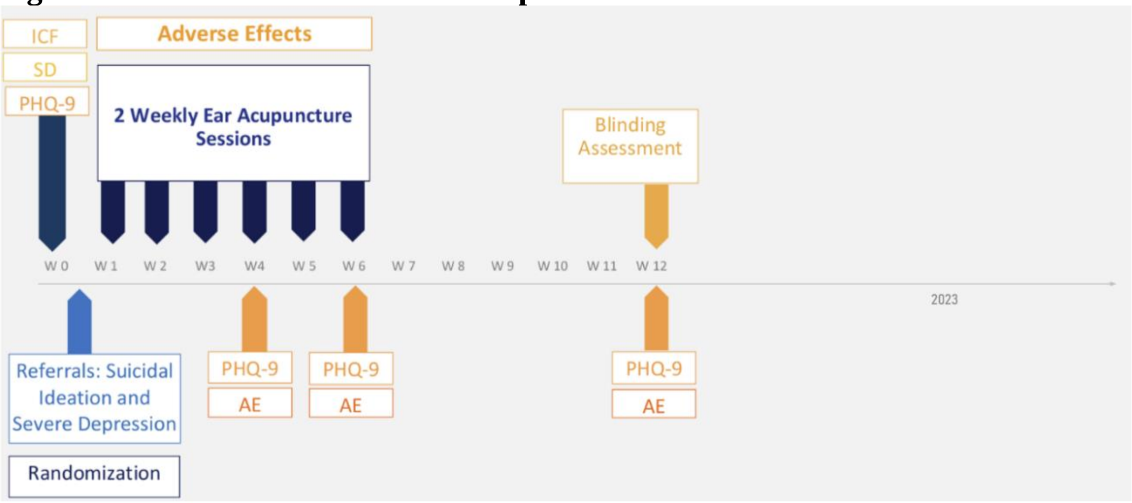

The protocol applicators will remove the auricular needles from the participants' ears from previous data collection interviews to ensure the blinding of the assessors, such as the adverse events questionnaire and the PHQ-9. Besides this procedure, study participants will be instructed to refrain from providing information about auricular acupuncture to the assessors, thus avoiding unmasking the intervention. The assessors will also be trained to avoid this bias. For the blinding assessment, participants will be asked to guess which treatment they received after three months of the study.

The protocol applicators will not know the participants' depression levels during the study. During the applications, communication with the participants will be limited to necessary explanations about the study to avoid potential biases through suggestive observations. These researchers will be trained to avoid such interference. Professionals with specific training in the field, with a specialization of 1,200 hours and a minimum of 10 years of experience, will be selected. These acupuncturists underwent extensive training in the study protocol and took theoretical and practical exams to assess uniformity in protocol application.

This study will follow the criteria of good clinical practice according to "The International Conference on Harmonisation of Technical Requirements for Registration of Pharmaceuticals for Human Use" from the International Conference on Harmonisation (ICH), including regular independent monitoring and the establishment of an independent data monitoring and safety committee. In addition to these guidelines, ethical principles advocated in the Declaration of Helsinki and the resolutions of the National Health Council related to research ethics, especially CNS Resolution 466/2012, will be followed. Interim assessments of the data collected during the study will be conducted to monitor the effectiveness and safety of the treatment, ensuring the participants' safety and the efficacy of the investigational treatment. This analysis will be conducted in a blinded and unbiased manner with statistical rigor to avoid misinterpretation.

8. Adherence

Participants who do not return for data collection assessments will be considered non-adherent to the study. Two non-consecutive absences will be allowed during the treatment sessions, as the literature suggests that auricular acupuncture has effects with weekly applications[44]. Participants who attend the assessments will be considered safe from follow-up.

9. Sample Size

The sample size was designed to detect a difference of 30% (experimental group 60%, and control group 30%) in the recovery of depressive symptoms (PHQ-9 <10) between the two groups. A minimum sample size of 36 participants per group is estimated, considering a two-tailed test, 80% power, and a significance level of 5%. An additional 10% was added to account for potential dropouts, resulting in 40 participants in each group, making a total of 80 participants [86].

10. Statistical analysis

An independent statistician hired for his/her experience in clinical trials will analyze the data in this clinical trial. This professional will be blinded to the treatment groups, ensuring impartiality in the data

analysis. Statistical procedures will also be employed to assess treatment safety and identify adverse events besides the statistical methods used to evaluate the effects of specific and non-specific auricular acupuncture in treating depression. Initially, a baseline comparison between the specific and non-specific auricular acupuncture groups will be conducted using Fisher's Exact Test. Subsequently, intention-to-treat and per-protocol analyses will be performed to compare the proportions of depression recovery and remission at different time points in the study (4 and 6 weeks and 3 months). These analyses will be carried out to evaluate the treatments' effectiveness in both groups. Fisher's Exact Test will be adopted to compare the proportions. The relative risk (RR) will be calculated to assess the difference in depressive symptoms between the two groups after 4 and 6 weeks and 3 months of intervention. This measure will determine the magnitude of the effect of specific auricular acupuncture in reducing depression symptoms compared to the control group.

The scores will be subjected to a normality test to assess the normality of the sample. If the assumption of normality is met, the Student's t-test will be used to compare means between the groups for two measures. However, if the assumption of normality is not met, the Wilcoxon-Mann-Whitney test will be employed to analyze the decrease in depression scores over time within both groups.

For comparisons involving three or more measures, the analysis of variance (ANOVA) followed by post-hoc tests will be performed if the data meet the assumption of normality. If not, the Kruskal-Wallis test will be used. In case of significant differences, pairwise comparisons will be conducted using appropriate non-parametric tests. These approaches will ensure robust statistical analysis, accounting for potential variations in data distribution. These tests will be followed by post-hoc tests to identify specific time points with significant differences. All analyses will be conducted with a 5% significance level.

A comprehensive sensitivity analysis with imputation of missing data will be conducted to achieve rigorous data analysis and ensure the reliability of our study outcomes. This analysis will serve as a critical component of our study design to address potential data gaps and enhance the robustness of our findings.

Multiple imputation methods will be systematically explored as part of this sensitivity analysis. These methods will include the widely-accepted LOCF (Last Observation Carried Forward), imputation using the GEE model, and the utilization of multiple imputation techniques through the EM (Expectation-Maximization Lag) algorithm. These approaches will be specifically applied to address missing data in categorical variables, ultimately assisting us in producing comprehensive and accurate results.

Furthermore, our analysis will employ the Generalized Estimating Equations (GEE) model, tailored to provide specific insights into the PHQ-9 depression scores. This model will be instrumental in quantifying the impact of several factors and variables on the participants' depression scores, thus allowing us to draw more precise conclusions regarding the efficacy of our interventions.

We aim to enhance the validity and reliability of our study's outcomes through this in-depth sensitivity analysis with robust imputation methods and the GEE model. This proactive approach will enable us to address and mitigate potential data limitations and variations, resulting in more comprehensive and insightful findings for the benefit of our study's scientific integrity and practical applications.

Relative and absolute frequencies and Fisher's Exact Test will be used to assess the safety of treatments and identify adverse events. These approaches will allow for a comprehensive analysis of the effects and adverse events of specific and non-specific auricular acupuncture in treating depression.

Moreover, Fisher's exact test will assess the success of blinding and the perceived treatment efficacy. The statistical analyses will be performed using the R program, a widely used open-source statistical programming language in medical research. The R program offers various statistical functions that enable robust and accurate analyses.

11. Ethics and dissemination

The recommendations of Resolution N° 466/12 of the National Health Council (CNS) will be adopted for this research, as it will involve the participation of human subjects. Participants will receive one of the two copies of the Informed Consent Form, ensuring compliance with the ethical principles outlined in Resolution N° 466/12.

Additionally, this research ensures the information confidentiality and anonymity, the return of research results to the subjects and society, and access to research results for participants. The researchers declare the absence of any interests related to the research outcomes. The research project has been submitted for evaluation to the Research Ethics Committee (CEP) of the School of Medicine of the University of São Paulo (FM-USP) and the Ethics Committee (CEP) of the University of Southern Santa Catarina. Data will only be collected after approval and stored on the REDCap server at the Hospital das Clínicas of FMUSP. All data will be electronically recorded and securely and accessibly stored on servers at HC-FMUSP for a minimum of five years after the completion of the research, per item XI of Resolution CNS N° 466/12.

Participants with severe depression or suicidal ideation will be excluded from the study due to the severity of their symptoms. These participants will be followed and medicated by a psychiatrist and researcher of the study according to their needs and referred to the university's Psychology Clinic for psychological treatment and the Natural Therapies clinic for complementary treatment.

11.1. Risk-benefit assessment

Understanding that, according to CNS Resolution N° 466/12, all human research entails various types and degrees of risks, this study anticipates minimal risks. The screening process using the PHQ-9 may be distressing for some participants during the recruitment phase. The nature of the questions may induce certain emotional feelings, including sadness or crying. Others may worry about the PHQ-9 results, suggesting depressive symptoms or requiring further specialized care. All research assessors will be trained in ways to minimize these feelings and how to handle such situations.

From screening to any of the follow-up contacts, researchers may come across severely depressed, suicidal, or individuals with other severe mental disorders. These participants will be excluded from the study analysis and referred to a psychiatrist who will take the necessary actions for each case.

Some participants may be concerned about the confidentiality of their data, primarily related to the potential exposure of opinions that could lead to possible embarrassment among peers. Care will be taken to maintain confidentiality and anonymity in order to minimize risks such as these.

The anticipated minimal risks refer to minor discomforts associated with auricular acupuncture application, such as mild pain at the auricular point and the possible appearance of other physical or emotional reactions due to the technique. Other risks that may be identified will be controlled.

Regarding benefits, research procedures during the recruitment phase may identify patients with depression and possibly at risk of suicide, who might not have been identified otherwise, ensuring specialized care for severe depression or suicidal ideation or a traditional treatment for participants in the experimental group.

This study will test a simple, cost-effective, non-pharmacological, and easy-to-apply intervention for depression. Therefore, it can generate significant public health gains toward improving access to complementary treatments and reducing the treatment gap for depressive disorders. The knowledge gained could be crucial in influencing the Brazilian collective and mental health policies agenda.

Furthermore, study results will be made available to participants via email or in any other way chosen by the participant after publication.

Therefore, this research is justified in its risk-benefit balance, as the potential benefits outweigh the possible risks. Additionally, we should underscore that auricular acupuncture is widely used globally. It is a technique with easy application, low cost, and minimal side effects.

11.2. From the process of informed consent

This study provides for an informed consent process to ensure due respect and human dignity. Therefore, researchers will contact participants in person or via telephone to extend the invitation to participate in the research. During this contact, the best time, conditions, and suitable location will be identified per the participant's preferences to ensure his/her privacy. Adequate time for reading, understanding, and reflection will be ensured. The informed consent form (ICF) will be designed to provide all necessary information in a language accessible to participants, which will be read and comprehended before granting their informed consent. The questionnaires will be answered only after signing the ICF.

11.3. About the Informed Consent Form

All Resolution CNS N° 466/12 recommendations were followed in preparing this form. The form was prepared in duplicate, with both copies signed by the researchers and participants. Participants retained the second copy with the respective protocol number. The Informed Consent Form also includes the contact information of the researchers, CEP-USP, and CEP-UNISUL.

References

1. Thombs BD, Bonardi O, Rice DB, et al. Curating evidence on mental health during COVID-19: A living systematic review. *J Psychosom Res*. 2020;133:110113. doi:10.1016/J.JPSYCHORES.2020.110113
2. Vindegaard N, Benros ME. COVID-19 pandemic and mental health consequences: Systematic review of the current evidence. *Brain Behav Immun*. 2020;89:531-542. doi:10.1016/J.BBI.2020.05.048
3. Kawohl W, Nordt C. COVID-19, unemployment, and suicide. *Lancet Psychiatry*. 2020;7(5):389-390. doi:10.1016/S2215-0366(20)30141-3
4. Beck A, Alford B. *Depressão: Causas e Tratamento.* 2nd ed. ArtMed; 2011.
5. World Health Organization. Depression and Other Common Mental Disorders: Global Health Estimates. *https://apps.who.int/iris/handle/10665/254610*. Published online 2017.
6. Prevalence of Depression Among Adults Aged 20 and Over: United States, 2013-2016 - PubMed. Accessed July 30, 2023. https://pubmed.ncbi.nlm.nih.gov/29638213/
7. Guo N, Robakis T, Miller C, Butwick A. Prevalence of Depression Among Women of Reproductive Age in the United States. *Obstetrics and gynecology*. 2018;131(4):671-679. doi:10.1097/AOG.0000000000002535
8. World mental health report: Transforming mental health for all. Accessed July 30, 2023. https://www.who.int/publications/i/item/9789240049338
9. American Psychiatric Association. *Manual Diagnóstico e Estatístico de Transtornos Mentais: DSM-5.* ArtMed; 2014.
10. Molendijk ML, Spinhoven P, Polak M, Bus BAA, Penninx BWJH, Elzinga BM. Serum BDNF concentrations as peripheral manifestations of depression: evidence from a systematic review and meta-analyses on 179 associations (N=9484). *Mol Psychiatry*. 2014;19(7):791-800. doi:10.1038/MP.2013.105
11. Bus BAA, Molendijk ML, Tendolkar I, et al. Chronic depression is associated with a pronounced decrease in serum brain-derived neurotrophic factors over time. *Mol Psychiatry*. 2015;20(5):602-608. doi:10.1038/MP.2014.83
12. Irwin MR, Miller AH. Depressive disorders and immunity: 20 years of progress and discovery. *Brain Behav Immun*. 2007;21(4):374-383. doi:10.1016/J.BBI.2007.01.010
13. Raison CL, Capuron L, Miller AH. Cytokines sing the blues: inflammation and the pathogenesis of depression. *Trends Immunol*. 2006;27(1):24-31. doi:10.1016/J.IT.2005.11.006
14. Simon NM, McNamara K, Chow CW, et al. A detailed examination of cytokine abnormalities in Major Depressive Disorder. *Eur Neuropsychopharmacol*. 2008;18(3):230-233. doi:10.1016/J.EURONEURO.2007.06.004
15. Barlow D, Durand M. *Psicopatologia: Uma Abordagem Integrada*. Cengage Learning; 2008.
16. American Psychiatric Association. *Diretrizes Para o Tratamento de Transtornos Psiquiátricos: Compêndio 2006.* ArtMed; 2008.
17. Cramer JA, Rosenheck R. Compliance with medication regimens for mental and physical disorders. *Psychiatr Serv*. 1998;49(2):196-201. doi:10.1176/PS.49.2.196
18. de Fátima Cunha M, de Cássia Gandini R. Adesão e não-adesão ao tratamento farmacológico para depressão. *Psicologia: Teoria e Pesquisa*. 2009;25(3):409-418. doi:10.1590/S0102-37722009000300015
19. Sackeim HA, Rush AJ, George MS, et al. Vagus nerve stimulation (VNS) for treatment-resistant depression: efficacy, side effects, and predictors of outcome. *Neuropsychopharmacology*. 2001;25(5):713-728. doi:10.1016/S0893-133X(01)00271-8
20. Moksnes KM, Ilner SO. Electroconvulsive therapy – efficacy and side-effects. *Tidsskrift for Den norske legeforening*. 2010;130(24):2460-2464. doi:10.4045/TIDSSKR.09.1102
21. Shiozawa P, da Silva ME, de Carvalho TC, Cordeiro Q, Brunoni AR, Fregni F. Transcutaneous vagus and trigeminal nerve stimulation for neuropsychiatric disorders: a systematic review. *Arq Neuropsiquiatr*. 2014;72(7):542-547. doi:10.1590/0004-282X20140061
22. Carreno FR, Frazer A. The Allure of Transcutaneous Vagus Nerve Stimulation as a Novel Therapeutic Modality. *Biol Psychiatry*. 2016;79(4):260-261. doi:10.1016/J.BIOPSYCH.2015.11.016
23. Wu C, Liu P, Fu H, et al. Transcutaneous auricular vagus nerve stimulation in treating major depressive disorder: A systematic review and meta-analysis. *Medicine*. 2018;97(52). doi:10.1097/MD.0000000000013845
24. De Matos e Souza FG. Tratamento da depressão. *Brazilian Journal of Psychiatry*. 1999;21(SUPPL. 1):18-23. doi:10.1590/S1516-44461999000500005

25. Luiz MT. Cultura contemporânea e medicinas alternativas: novos paradigmas em saúde no fim do século XX. *Physis: Revista de Saúde Coletiva*. 1997;7(1):13-43. doi:10.1590/S0103-73311997000100002
26. World Health Organization. WHO Traditional Medicine Strategy 2014-2023. *https://www.who.int/publications/i/item/9789241506096*. Published online 2013.
27. World Health Organization. WHO global report on traditional and complementary medicine 2019. *https://apps.who.int/iris/handle/10665/312342*. Published online 2019.
28. Harris PE, Cooper KL, Relton C, Thomas KJ. Prevalence of complementary and alternative medicine (CAM) use by the general population: a systematic review and update. *Int J Clin Pract*. 2012;66(10):924-939. doi:10.1111/J.1742-1241.2012.02945.X
29. Peltzer K, Pengpid S. Prevalence and Determinants of Traditional, Complementary and Alternative Medicine Provider Use among Adults from 32 Countries. *Chin J Integr Med*. 2018;24(8):584-590. doi:10.1007/S11655-016-2748-Y
30. Brasil. Ministério da Saúde. Secretaria de Atenção à Saúde. Departamento de Saúde da Família. Relatório de Monitoramento Nacional das Práticas Integrativas e Complementares nos Sistemas de Informação em Saúde. Published online 2020.
31. Brasil. Ministério da Saúde. Secretaria de Atenção à Saúde. Departamento de Atenção Básica. Política Nacional de Práticas Integrativas e Complementares no SUS - PNPIC. Published online 2006.
32. Sabbag SHF, Nogueira BMR, Callis ALL De, et al. Origem e desenvolvimento da Naturologia no Brasil: aspectos conceituais, históricos e políticos. *Cad naturol terap complem*. 2017;6(11):59-68. doi:10.19177/CNTC.V6E11201759-68
33. World Health Organization. Atlas de Medicina Complementares e Tradicional. Published online 2014.
34. About us | Cochrane Complementary Medicine. Accessed July 30, 2023. https://cam.cochrane.org/
35. Maciocia G. *Diagnóstico Na Medicina Chinesa, Um Guia Geral*. Roca; 2005.
36. Chung S, Cha S, Lee SY, Park JH, Lee S. The five elements of the cell. *Integr Med Res*. 2017;6(4):452-456. doi:10.1016/J.IMR.2017.10.002
37. O'Brien K, Weber D. Insomnia in Chinese Medicine: The Heart of the Matter. *J Altern Complement Med*. 2016;22(9):684-694. doi:10.1089/ACM.2016.0044
38. Ju D, Liu M, Zhao H, et al. Mechanisms of "kidney governing bones" theory in traditional Chinese medicine. *Front Med*. 2014;8(3):389-393. doi:10.1007/S11684-014-0362-Y
39. The paradox of the unity and duality of the kidneys according to Chinese medicine: kidney essence, yin, yang, qi, the mingmen--their origins, relationships, functions and manifestations - PubMed. Accessed July 30, 2023. https://pubmed.ncbi.nlm.nih.gov/10729971/
40. Maciocia G. *Os Fundamentos Da Medicina Chinesa*. 3rd ed. Roca; 2017.
41. Rodrigues DM de O. Medicina tradicional chinesa: contextualização e utilização no ocidente. *Cadernos de Naturologia e Terapias Complementares*. 2015;4(7):9-10. doi:10.19177/CNTC.V4E720159-10
42. Macpherson H, Elliot B, Hopton A, Lansdown H, Richmond S. Acupuncture for Depression: Patterns of Diagnosis and Treatment within a Randomised Controlled Trial. *Evid Based Complement Alternat Med*. 2013;2013. doi:10.1155/2013/286048
43. World Health Organization. Benchmarks for training in traditional /complementary and alternative medicine: benchmarks for training in traditional Chinese medicine. *https://apps.who.int/iris/handle/10665/44353*. Published online 2010.
44. Dal Mas WD. *Acupuntura Auricular Na Doutrina Brasileira*. Roca; 2004.
45. Definition of ear acupuncture - NCI Dictionary of Cancer Terms - NCI. Accessed July 30, 2023. https://www.cancer.gov/publications/dictionaries/cancer-terms/def/ear-acupuncture
46. Hou PW, Hsu HC, Lin YW, Tang NY, Cheng CY, Hsieh CL. The History, Mechanism, and Clinical Application of Auricular Therapy in Traditional Chinese Medicine. *Evid Based Complement Alternat Med*. 2015;2015. doi:10.1155/2015/495684
47. La Marca R, Nedeljkovic M, Yuan L, Maercker A, Ehlert U. Effects of auricular electrical stimulation on vagal activity in healthy men: evidence from a three-armed randomized trial. *Clin Sci (Lond)*. 2010;118(8):537-546. doi:10.1042/CS20090264
48. Peuker ET, Filler TJ. The nerve supply of the human auricle. *Clin Anat*. 2002;15(1):35-37. doi:10.1002/CA.1089
49. Corrêa HP, Moura C de C, Azevedo C, Bernardes MFVG, Mata LRFP da, Chianca TCM. Efeitos da auriculoterapia sobre o estresse, ansiedade e depressão em adultos e idosos: revisão

sistemática. *Revista da Escola de Enfermagem da USP*. 2020;54:e03626. doi:10.1590/S1980-220X2019006703626

50. Kreuzer PM, Landgrebe M, Resch M, et al. Feasibility, safety and efficacy of transcutaneous vagus nerve stimulation in chronic tinnitus: an open pilot study. *Brain Stimul*. 2014;7(5):740-747. doi:10.1016/J.BRS.2014.05.003
51. Kraus T, Hösl K, Kiess O, Schanze A, Kornhuber J, Forster C. BOLD fMRI deactivation of limbic and temporal brain structures and mood enhancing effect by transcutaneous vagus nerve stimulation. *J Neural Transm (Vienna)*. 2007;114(11):1485-1493. doi:10.1007/S00702-007-0755-Z
52. Fang J, Rong P, Hong Y, et al. Transcutaneous Vagus Nerve Stimulation Modulates Default Mode Network in Major Depressive Disorder. *Biol Psychiatry*. 2016;79(4):266-273. doi:10.1016/J.BIOPSYCH.2015.03.025
53. Jonas WB, Bellanti DM, Paat CF, et al. A Randomized Exploratory Study to Evaluate Two Acupuncture Methods for the Treatment of Headaches Associated with Traumatic Brain Injury. *Med Acupunct*. 2016;28(3):113. doi:10.1089/ACU.2016.1183
54. Sun M, Lu Q, Zeng X, Bian X. [Acupressure combined with press needles for prevention of gastroscopy-induced nausea and vomiting]. *Zhongguo Zhen Jiu*. 2016;36(11):1131-1134. doi:10.13703/J.0255-2930.2016.11.003
55. Kurebayashi LFS, da Silva MJP. Eficácia da auriculoterapia chinesa para o estresse em equipe de enfermagem: ensaio clínico randomizado. *Rev Lat Am Enfermagem*. 2014;22(3):371-378. doi:10.1590/0104-1169.3239.2426
56. [Clinical observation of depression after breast cancer operation treated with auricular point sticking therapy] - PubMed. Accessed July 31, 2023. https://pubmed.ncbi.nlm.nih.gov/26255515/
57. Set T, Cayir Y, Guven Pirim AB. Effects of ear acupuncture therapy for obesity on the depression of obese women. *Acupuncture in medicine : journal of the British Medical Acupuncture Society*. 2014;32(5):427-429. doi:10.1136/ACUPMED-2014-010626
58. [Comparative study on effects between electroacupuncture and auricular acupuncture for methamphetamine withdrawal syndrome] - PubMed. Accessed July 30, 2023. https://pubmed.ncbi.nlm.nih.gov/24843957/
59. Fu W Bin, Fan L, Zhu XP, et al. Depressive neurosis treated by acupuncture for regulating the liver--a report of 176 cases. *J Tradit Chin Med*. 2009;29(2):83-86. doi:10.1016/S0254-6272(09)60037-6
60. Afrasiabi J, McCarty R, Hayakawa J, et al. Effects of Acupuncture and Acupressure on Burnout in Health Care Workers: A Randomized Trial. *J Trauma Nurs*. 2021;28(6):350-362. doi:10.1097/JTN.0000000000000614
61. King HC, Spence DL, Hickey AH, Sargent P, Elesh R, Connelly CD. Auricular acupuncture for sleep disturbance in veterans with post-traumatic stress disorder: a feasibility study. *Mil Med*. 2015;180(5):582-590. doi:10.7205/MILMED-D-14-00451
62. Cha NH, Park YK, Sok SR. Effects of Auricular Acupressure Therapy on Stress and Sleep Disturbance of Middle-Aged Women in South Korea. *Holist Nurs Pract*. 2017;31(2):102-109. doi:10.1097/HNP.0000000000000197
63. Kessler RC, Soukup J, Davis RB, et al. The use of complementary and alternative therapies to treat anxiety and depression in the United States. *Am J Psychiatry*. 2001;158(2):289-294. doi:10.1176/APPI.AJP.158.2.289
64. Jorm AF, Korten AE, Jacomb PA, Christensen H, Rodgers B, Pollitt P. "Mental health literacy": a survey of the public's ability to recognise mental disorders and their beliefs about the effectiveness of treatment. *Med J Aust*. 1997;166(4):182-186. doi:10.5694/J.1326-5377.1997.TB140071.X
65. Jorm AF, Medway J, Christensen H, Korten AE, Jacomb PA, Rodgers B. Public beliefs about the helpfulness of interventions for depression: effects on actions taken when experiencing anxiety and depression symptoms. *Aust N Z J Psychiatry*. 2000;34(4):619-626. doi:10.1080/J.1440-1614.2000.00761.X
66. Yang Y, Wen J, Hong J. The Effects of Auricular Therapy for Cancer Pain: A Systematic Review and Meta-Analysis. *Evid Based Complement Alternat Med*. 2020;2020. doi:10.1155/2020/1618767
67. Geib J, Rieger MA, Joos S, Eschweiler GW, Dresler T, Metzger FG. Introduction of auricular acupuncture in elderly patients suffering from major depression: protocol of a mixed methods feasibility study. *Biomed Res Int*. 2015;2015. doi:10.1155/2015/678410

68. White A, Hayhoe S, Hart A, Ernst E. Survey of adverse events following acupuncture (SAFA): a prospective study of 32,000 consultations. *Acupuncture in medicine : journal of the British Medical Acupuncture Society*. 2001;19(2):84-92. doi:10.1136/AIM.19.2.84
69. MacPherson H, Thomas K, Walters S, Fitter M. The York acupuncture safety study: prospective survey of 34 000 treatments by traditional acupuncturists. *BMJ*. 2001;323(7311):486-487. doi:10.1136/BMJ.323.7311.486
70. Leung MCP, Yip KK, Ho YS, Siu FKW, Li WC, Garner B. Mechanisms underlying the effect of acupuncture on cognitive improvement: a systematic review of animal studies. *J Neuroimmune Pharmacol*. 2014;9(4):492-507. doi:10.1007/S11481-014-9550-4
71. Huang W, Pach D, Napadow V, et al. Characterizing acupuncture stimuli using brain imaging with FMRI--a systematic review and meta-analysis of the literature. *PLoS One*. 2012;7(4). doi:10.1371/JOURNAL.PONE.0032960
72. Liu P, Qin W, Zhang Y, et al. Combining spatial and temporal information to explore function-guide action of acupuncture using fMRI. *J Magn Reson Imaging*. 2009;30(1):41-46. doi:10.1002/JMRI.21805
73. Hamilton JP, Farmer M, Fogelman P, Gotlib IH. Depressive Rumination, the Default-Mode Network, and the Dark Matter of Clinical Neuroscience. *Biol Psychiatry*. 2015;78(4):224. doi:10.1016/J.BIOPSYCH.2015.02.020
74. Smith CA, Armour M, Lee MS, Wang LQ, Hay PJ. Acupuncture for depression. *Cochrane Database of Systematic Reviews*. 2018;2018(3). doi:10.1002/14651858.CD004046.PUB4/MEDIA/CDSR/CD004046/IMAGE_N/NCD004046-CMP-005-07.PNG
75. Santos IS, Tavares BF, Munhoz TN, et al. Sensibilidade e especificidade do Patient Health Questionnaire-9 (PHQ-9) entre adultos da população geral. *Cad saúde pública*. 2013;29(8):1533-1543. doi:10.1590/S0102-311X2013001200006
76. Löwe B, Unützer J, Callahan CM, Perkins AJ, Kroenke K. Monitoring depression treatment outcomes with the patient health questionnaire-9. *Med Care*. 2004;42(12):1194-1201. doi:10.1097/00005650-200412000-00006
77. Ell K, Unützer J, Aranda M, Gibbs NE, Lee PJ, Xie B. Managing depression in home health care: a randomized clinical trial. *Home Health Care Serv Q*. 2007;26(3):81-104. doi:10.1300/J027V26N03_05
78. Kroenke K, Spitzer RL, Williams JBW. The PHQ-9: validity of a brief depression severity measure. *J Gen Intern Med*. 2001;16(9):606-613. doi:10.1046/J.1525-1497.2001.016009606.X
79. Yates Coley R, Boggs JM, Beck A, Hartzler AL, Simon GE. Defining Success in Measurement-Based Care for Depression: A Comparison of Common Metrics. *Psychiatr Serv*. 2020;71(4):312-318. doi:10.1176/APPI.PS.201900295
80. Focks C. *Atlas de Acupuntura: Com Sequência de Fotos e Ilustrações, Textos Didáticos e Indicações Clínicas*. Manole; 2005.
81. Zhang CS, Yang AW, Zhang AL, May BH, Xue CC. Sham Control Methods Used in Ear-Acupuncture/Ear-Acupressure Randomized Controlled Trials: A Systematic Review. *Journal of Alternative and Complementary Medicine*. 2014;20(3):147. doi:10.1089/ACM.2013.0238
82. Prado JM do. *Aplicação Da Acupuntura Auricular Verdadeira e Sham No Tratamento Do Estresse Em Enfermeiros*. Escola de Enfermagem da Universidade de São Paulo; 2014.
83. Dincer F, Linde K. Sham interventions in randomized clinical trials of acupuncture--a review. *Complement Ther Med*. 2003;11(4):235-242. doi:10.1016/S0965-2299(03)00124-9
84. Zhong LLD, Kun W, Lam TF, et al. The combination effects of body acupuncture and auricular acupressure compared to sham acupuncture for body weight control: study protocol for a randomized controlled trial. *Trials*. 2016;17(1). doi:10.1186/S13063-016-1458-2
85. Kwon OS, Kim J, Choi KH, Ryu Y, Park JE. Trends in deqi research: a text mining and network analysis. *Integr Med Res*. 2018;7(3):231-237. doi:10.1016/J.IMR.2018.02.007
86. Browner W, Newman T, Hulley S. Estimando o tamanho da amostra e o poder estatístico: aplicações e exemplos. In: Huller SG, ed. *Delineando a Pesquisa Clínica: Uma Abordagem Epidemiológica*. 3rd ed. ArtMed; 2008.
