# Supplementary material for: Efficacy and Safety of Auricular Acupuncture for Depression: A Randomized Clinical Trial
Source: JAMA Netw Open. 2023 Nov 30;6(11):e2345138. doi: 10.1001/jamanetworkopen.2023.45138 (PMC10690462; doi:10.1001/jamanetworkopen.2023.45138)
Supplement: Supplement 2. — eTable 1. Inclusion and Exclusion Criteria eFigure 1. Data Collection Procedures eFigure 2. Points Applied in the Auricular Pavilion of the Specific Auricular Acupuncture (SA) Group eTable 2. Universal Nomenclature, Location, and Indications of the Points Used in the Groups eFigure 3. Points Applied in the Auricular Pavilion of the Nonspecific Auricular Acupuncture (NSA) Group eTable 3. Primary and Secondary Outcomes of the Study eTable 4. Loss to Follow-Up Throughout the Study eTable 5. Baseline Depression Score Analysis for Participants Who Did Not Complete the Study eTable 6. Secondary Outcomes of Depression Scores by Intention-to-Treat Analysis eTable 7. Secondary Efficacy Outcomes in Depression Recovery and Remission by Modified Intent-to-Treat Analysis eTable 8. Secondary Outcomes of Depression Scores by Modified Intent-to-Treat Analysis eFigure 4. Median Depression Scores by Modified Intent-to-Treat Analysis eTable 9. Sensitivity Analysis for Depression Recovery With Missing Data Imputation eTable 10. Sensitivity Analysis for Symptom Remission With Missing Data Imputation eTable 11. Sensitivity Analysis for Depression Score With Missing Data Imputation eTable 12. Adjustment of the Generalized Estimating Equations (GEE) Model eTable 13. Blinding Assessment and Perception of Treatment Efficacy [file jamanetwopen-e2345138-s002.pdf]

Supplemental Online Content

de Oliveira Rodrigues DM, Menezes PR, Machado Ribeiro Silotto A, et al. Efficacy and safety of auricular acupuncture for depression: a randomized clinical trial. *JAMA Netw Open*. 2023;6(11):e2345138. doi:10.1001/jamanetworkopen.2023.45138

eTable 1. Study Inclusion and Exclusion Criteria

eFigure 1. Data Collection Procedures

eFigure 2. Points Applied In The Auricular Pavilion of the Specific Auricular Acupuncture (SA) Group

eTable 2. Universal Nomenclature, Location, and Indications of the Points Used in the Groups

eFigure 3. Points Applied in the Auricular Pavilion of the Nonspecific Auricular Acupuncture (NSA) Group

eTable 3. Primary and Secondary Outcomes of the Study

eTable 4. Loss to Follow-Up Throughout the Study

eTable 5. Baseline Depression Score Analysis for Participants Who Did Not Complete the Study

eTable 6. Secondary Outcomes of Depression Scores by Intention-to-Treat Analysis

eTable 7. Secondary Efficacy Outcomes in Depression Recovery and Remission by Modified Intent-to-Treat Analysis

eTable 8. Secondary Outcomes of Depression Scores by Modified Intent-to-Treat Analysis

eFigure 4. Median Depression Scores by Modified Intent-to-Treat Analysis

eTable 9. Sensitivity Analysis for Depression Recovery With Missing Data Imputation

eTable 10. Sensitivity Analysis for Symptom Remission With Missing Data Imputation

eTable 11. Sensitivity Analysis for Depression Score with Missing Data Imputation

eTable 12. Adjustment of the Generalized Estimating Equations (GEE) Model

eTable 13. Blinding Assessment and Perception of Treatment Efficacy

This supplemental material has been provided by the authors to give readers additional information about their work.

© 2023 de Oliveira Rodrigues DM et al. *JAMA Network Open.*

eTable 1. Study Inclusion and Exclusion Criteria

**ELIGIBILITY**	The study's eligibility was assessed in a pre-screening conducted in a dedicated room, ensuring participant integrity, confidentiality, and anonymity. The following inclusion and exclusion criteria were employed for the study.
**INCLUSION CRITERIA**	• Age between 18 and 50 years;<br>• Attainment of minimum PHQ-9 scores for moderate/moderately severe depression;<br>• Availability during the session timings.
**EXCLUSION CRITERIA**	• Use of complementary therapies for a minimum of 3 months;<br>• Risk of suicidal ideation assessed through question 9 of the PHQ-9;<br>• Severe depression assessed via PHQ-9 scores;<br>• Prior application of auricular acupuncture;<br>• Pregnancy;<br>• Menopause;<br>• Allergy to metals and micropores;<br>• Healthcare students with a history of complementary therapies in the curriculum;<br>• Individuals with scheduled vacations during the data collection period;<br>• Lack of accessible earlobe areas due to mutilation, cartilage deformation, earring piercings, or other artifacts hindering the application of auricular technique at the specific point.

© 2023 de Oliveira Rodrigues DM et al. *JAMA Network Open.*

eFigure 1. Data Collection Procedures

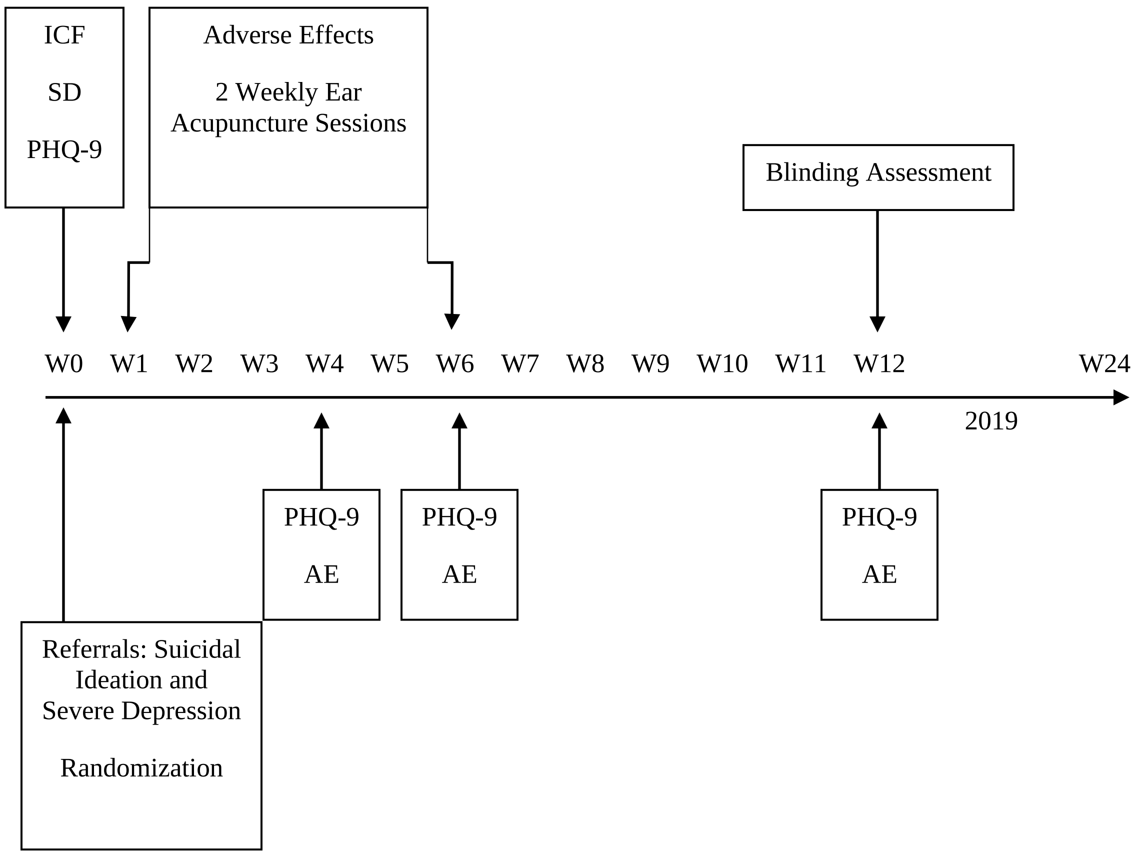

© 2023 de Oliveira Rodrigues DM et al. *JAMA Network Open.*

eFigure 2. Points Applied in the Auricular Pavilion of the Specific Auricular Acupuncture (SA) Group

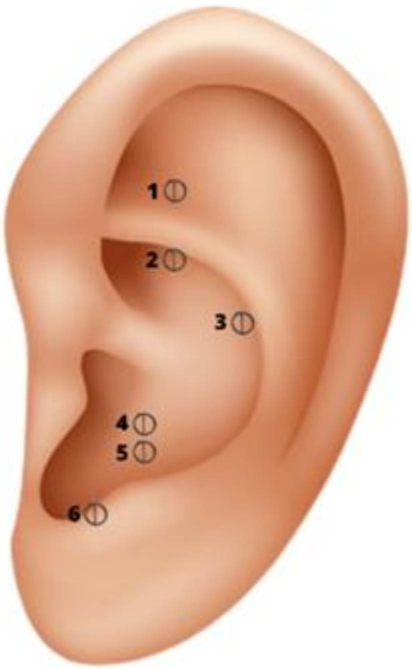

1)Shenmen / TF4 Shenmen

2) Kidney / CO10 Shen

3) Liver / CO12 Gan

4) Heart / CO15 Xin

5) Lung / CO14 Fei

6) Subcortex / AT4 Pizhixia

© 2023 de Oliveira Rodrigues DM et al. *JAMA Network Open.*

eTable 2. Universal Nomenclature, Location, and Indications of the Points Used in the Groups

Group	Ear Acupuncture Point / Universal and Chinese Nomenclature	Anatomical Location	Indications
**Specific AA (SA)**	*Shenmen / TF4 Shenmen*	Upper portion of the apex of the triangular fossa, closer to the upper branch of the antihelix.	Analgesic and anti-inflammatory action. It is the most crucial anesthesia point on the ear.
	Subcortex / *AT4 Pizhixia*	Inner and anterior faces of the antitragus	Rebalances nervous, digestive, and cardiovascular functions
	Heart / *CO15 Xin*	In the center of the cavum conchae	Used for cardiac complaints such as angina, cardiac rhythm disorders
	Lung / *CO14 Fei*	Above and below the heart point	Assists with respiratory tract diseases, indicated for nicotine dependence and skin conditions.
	Liver / *CO12 Gan*	In the cymba conchae, above the root of the helix, close to the antihelix.	Used for liver-related issues, intercostal neuralgia, and ocular conditions.
	Kidney / *CO10 Shen*	In the cymba conchae, in a groove below the start of the inferior crus.	Used for issues in the urinary and genital systems. Assists with tinnitus, auditory conditions, and sleep disorders.
**Non-specific AA (NSA)**	External Ear / *LO6 Neier*	In the depression between the tragus's upper end and the helix's ascending spine.	Used for inflammatory changes in the external ear. It assists with tinnitus, hearing loss, and temporomandibular joint pain.
	Cheek/Face / *LO5 - LO6 Mianjia*	In an oval portion in the region of the 2nd, 3rd, 5th, and 6th quadrants.	Used for myofascial spasms, facial paresis, and trigeminal neuralgia.
	4 Non-specific points in the Helix region / *HX9* Lunyi; *HX10* Lunar; *HX11* Lunsan; *HX12* Lunsi	On the margin of the auricle, between the helix tubercle and the apex, and between the tubercle and the lower lobe point.	Used for thirst control.

© 2023 de Oliveira Rodrigues DM et al. *JAMA Network Open.*

eFigure 3. Points Applied in the Auricular Pavilion of the Nonspecific Auricular Acupuncture (NSA) Group

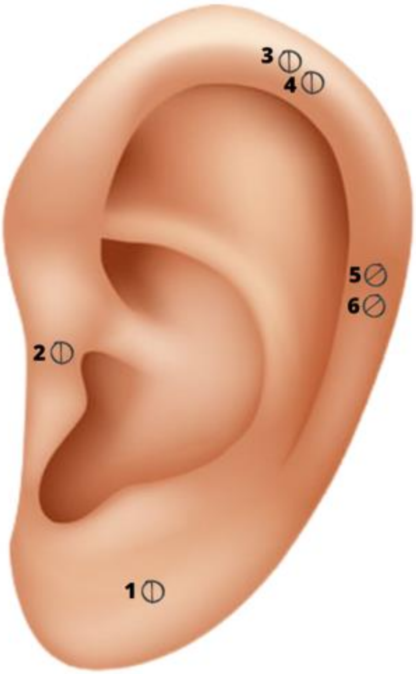

1)Cheek/Face / LO5 - LO6 Mianjia

2) External Ear / LO6 Neier

3-6)4 Non-specific points in the Helix region / HX9 Lunyi; HX10 Lunar; HX11 Lunsan; HX12 Lunsi

© 2023 de Oliveira Rodrigues DM et al. *JAMA Network Open.*

eTable 3. Primary and Secondary Outcomes of the Study

**PRIMARY OUTCOME**	The primary outcome was the difference in the proportion of participants who showed an improvement of 50% or more in depressive symptoms between the groups, as assessed by the PHQ-9 three months after inclusion. A follow-up score after 3 months showed a 50% improvement compared to baseline value was considered as a success or recovery of symptoms.
**SECONDARY OUTCOMES**<br>The secondary outcomes of the study were included to enrich the understanding of the primary outcome and to evaluate different aspects related to depressive symptoms and the auricular acupuncture intervention.	• Difference in the proportion of participants who showed an improvement of 50% or more in depressive symptoms between the groups, as assessed by the PHQ-9, at assessments conducted after four and six weeks of study inclusion.<br>• Difference in the proportion of participants who achieved a score lower than 5 on the PHQ-9, indicating symptom remission between the groups, as assessed at assessments conducted after four, six weeks, and three months of study inclusion.<br>• Differences in depression scores assessed by the PHQ-9 at baseline, the end of the fourth and sixth weeks of auricular acupuncture application, and also three months after inclusion between the groups.<br>• Difference in the proportion of adverse events reported by participants through the PHQ-9 after four, six weeks, and three months of study inclusion.<br>• Difference in the proportion of adverse effects of auricular acupuncture throughout the sessions between the groups.<br>• Evaluation of blinding and perceived efficacy between the groups.

© 2023 de Oliveira Rodrigues DM et al. *JAMA Network Open.*

eTable 4. Loss to Follow-Up Throughout the Study

Attrition Time	Specific Auricular Acupuncture (SA) (N=37)		Non-specific Auricular Acupuncture (NSA) (N=37)		p-value [a]
**4 Weeks – N (%)**	09	24.3	08	21.6	>0.99
**6 Weeks – N (%)**	02	5.4	03	8.1	>0.99
**3 Months – N (%)**	02	5.4	03	8.1	>0.99
**Total**	**13**	**35.1**	**14**	**37.8**	>0.99

[a] Statistical significance was calculated with Fisher exact test.

eTable 5. Baseline Depression Score Analysis for Participants Who Did Not Complete the Study

VARIABLES	Specific Auricular Acupuncture (SA) (N=37)	Non-specific Auricular Acupuncture (NSA) (N=37)	p-value [a]
**Depression Score at baseline**			
Median (Q1 - Q3)	14.0 (12.0 - 16.0)	17.0 (15.0 - 18.0)	0.06

eTable 6. Secondary Outcomes of Depression Scores by Intention-to-Treat Analysis

VARIABLES	Specific Auricular Acupuncture (SA)	Non-specific Auricular Acupuncture (NSA)	p-value [a]
**Depression Score at baseline (N = 74)**			
Median (Q1 - Q3)	14.0 (12.0 - 16.0)	16.0 (14.0 - 18.0)	0.03
**Depression Score 4 weeks after baseline (N = 57)**			
Median (Q1 - Q3)	7.5 (4.0 - 11.0)	10.0 (6.0 - 13.0)	0.18
**Depression Score 6 weeks after baseline (N = 51)**			
Median (Q1 - Q3)	9.0 (4.3 - 13.0)	9.0 (6.0 - 16.0)	0.51
**Depression Score 3 months after baseline (N = 47)**			
Median (Q1 - Q3)	5.5 (3.8 - 11.0)	9.0 (6.5 - 14.0)	0.09

[a] Wilcoxon-Mann-Whitney Unpaired Test
Legend: Q1: first quartile; Q3: third quartile

© 2023 de Oliveira Rodrigues DM et al. *JAMA Network Open.*

eTable 7. Secondary Efficacy Outcomes in Depression Recovery and Remission by Modified Intent-to-Treat Analysis

VARIABLES	Specific Auricular Acupuncture (SA) (N=24)	p-value [b]	Non-specific Auricular Acupuncture (NSA) (N=23)	p-value [b]	p-value [a]
**Depression recovery at 4 weeks compared to baseline, n(%)**					
Yes	10 (42)		6 (27)		0.36
**Depression recovery at 6 weeks compared to baseline. n(%)**					
Yes	9 (37)	0.12	9 (41)	0.28	>0.99
**Depression recovery at 3 months compared to baseline. n(%)**					
Yes	14 (58)		9 (41)		0.38
**Symptom remission at 4 weeks compared to baseline. n(%)**					
Yes	6 (25)		4 (18)		0.72
**Symptom remission at 6 weeks compared to baseline. n(%)**					
Yes	6 (25)	0.06	5 (23)	0.54	>0.99
**Symptom remission at 3 months compared to baseline. n(%)**					
Yes	**11 (46)**		**3 (14)**		**0.03**

[a] Fisher's Exact Test
[b] Cochran's Q Test

© 2023 de Oliveira Rodrigues DM et al. *JAMA Network Open.*

eTable 8. Secondary Outcomes of Depression Scores by Modified Intent-to-Treat Analysis

VARIABLES	Specific Auricular Acupuncture (SA) (N=24)	p-value [b]	Non-specific Auricular Acupuncture (NSA) (N=23)	p-value [b]	p-value [a]
**Depression Score at baseline**					
Median (Q1 - Q3)	14.0 (12.0 - 17.0)		15.0 (13.2 - 17.0)		0.20
**Depression Score 4 weeks after baseline**					
Median (Q1 - Q3)	7.5 (4.8 - 11.0)	**<0.001**	10.0 (7.0 - 12.8)	**<0.001**	0.20
**Depression Score 6 weeks after baseline**					
Median (Q1 - Q3)	9.0 (4.8 - 13.0)	**<0.001**	9.0 (6.3 - 15.2)	**0.001**	0.70
**Depression Score 3 months after baseline**					
Median (Q1 – Q3)	5.5 (3.8 – 11.0)	**<0.001**	9.0 (6.3 – 14.5)	**<0.001**	0.08

[a] Wilcoxon-Mann-Whitney Unpaired
[b] Test Friedman's ANOVA followed by post-hoc test
Legend: Q1: first quartile; Q3: third quartile

© 2023 de Oliveira Rodrigues DM et al. *JAMA Network Open.*

eFigure 4. Median Depression Scores by Modified Intent-to-Treat Analysis

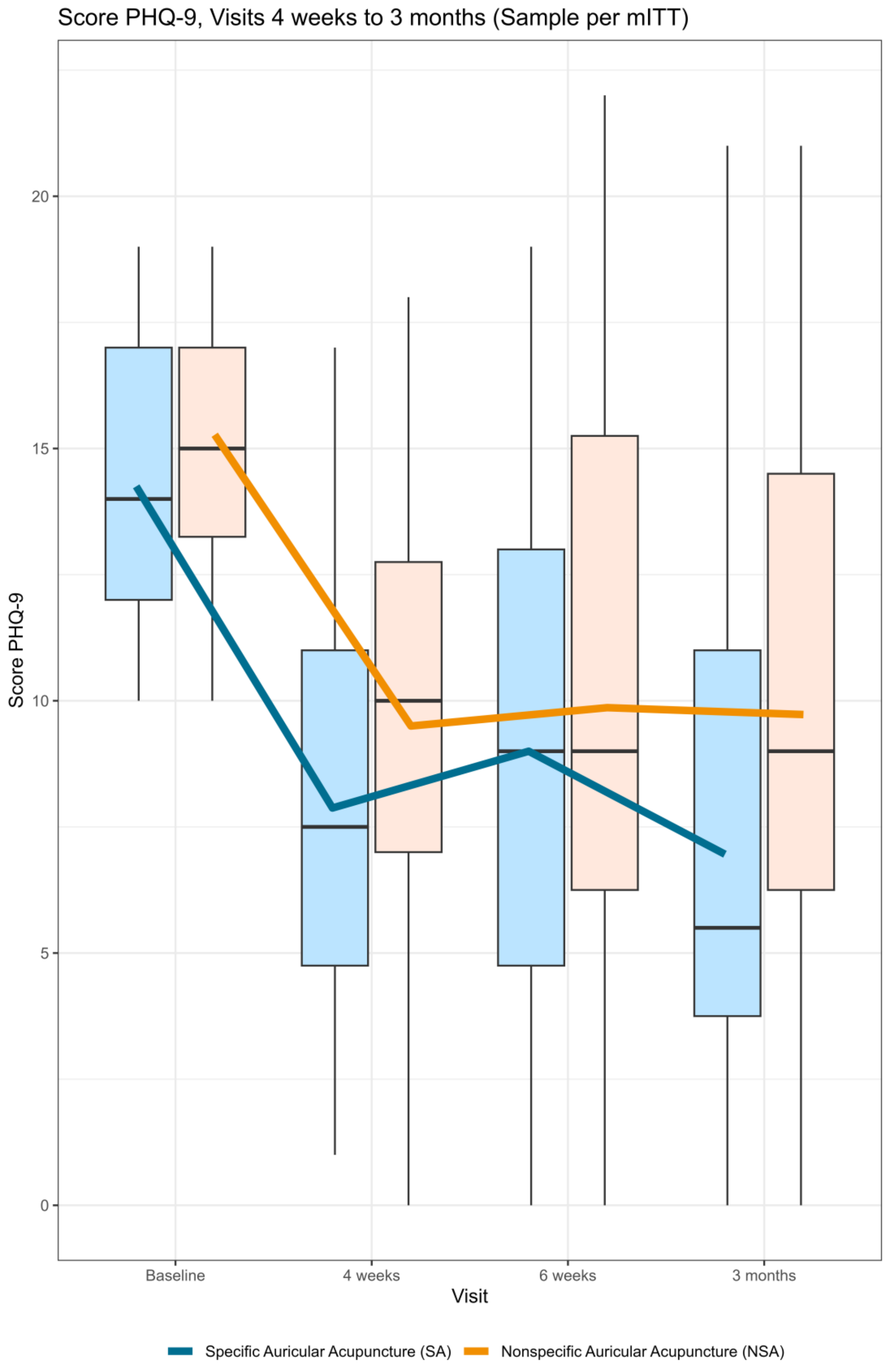

© 2023 de Oliveira Rodrigues DM et al. *JAMA Network Open.*

eTable 9. Sensitivity Analysis for Depression Recovery With Missing Data Imputation

Depression Recovery	Specific Auricular Acupuncture (SA) (N=37)	Non-specific Auricular Acupuncture (NSA) (N=37)	p-value [a]
**Last Observation Carried Forward**			
4 weeks	12 (32)	9 (24)	0.60
6 weeks	10 (27)	12 (32)	0.80
3 months	15 (40)	12 (32)	0.63
**Generalized Estimating Equations**			
4 weeks	17 (46)	10 (27)	0.15
6 weeks	12 (32)	10 (27)	0.80
3 months	19 (51)	12 (32)	0.16
**Expectation-Maximization Lag**			
4 weeks	12 (32)	10 (27)	0.80
6 weeks	16 (43)	15 (40)	>0.99
3 months	18 (49)	15 (40)	0.64

[a] Fisher's Exact Test

eTable 10. Sensitivity Analysis for Symptom Remission With Missing Data Imputation

Symptom remission	Specific Auricular Acupuncture (SA) (N=37)	Non-specific Auricular Acupuncture (NSA) (N=37)	p-value [a]
**Last Observation Carried Forward**			
4 weeks	8 (22)	5 (13)	0.54
6 weeks	7 (19)	6 (16)	>0.99
3 months	**12 (32)**	**4 (10)**	**0.04**
**Generalized Estimating Equations**			
4 weeks	8 (22)	5 (13)	0.54
6 weeks	7 (199)	5 (13)	0.75
3 months	**11 (30)**	**3 (8)**	**0.03**
**Expectation-Maximization Lag**			
4 weeks	8 (22)	6 (16)	0.76
6 weeks	9 (24)	7 (19)	0.77
3 months	**13 (35)**	**5 (13)**	**0.05**

[a] Fisher's Exact Test

© 2023 de Oliveira Rodrigues DM et al. *JAMA Network Open.*

Table 11. Sensitivity Analysis for Depression Score with Missing Data Imputation

Depression Score - Median (Q1 - Q3)	Specific Auricular Acupuncture (SA) (N=37)		Non-specific Auricular Acupuncture (NSA) (N=37)		p-value [a]
**Generalized Estimating Equations**					
Baseline	**14.0**	**(12.0 - 16.0)**	**16.0**	**(14.0 - 18.0)**	**0.02**
4 weeks	**7.9**	**(5.0 - 10.0)**	**9.5**	**(7.0 - 12.0)**	**0.04**
6 weeks	8.7	(6.0 - 10.0)	10.2	(8.0 - 10.2)	0.09
3 months	**7.5**	**(4.0 - 8.0)**	**9.1**	**(8.0 - 10.0)**	**0.01**

[a] Wilcoxon-Mann-Whitney Unpaired Test
Legend: Q1: first quartile; Q3: third quartile

eTable 12. Adjustment of the Generalized Estimating Equations (GEE) Model

Variable	Estimative	Standard Error	Wald Est	p-value
Intercept	13.98	0.514	740.50	<0.001
Non-specific Auricular Acupuncture (NSA)	1.57	0.682	5.30	0.02
4 weeks	-6.08	0.753	65.30	<0.001
6 weeks	-5.32	0.904	34.70	<0.001
3 months	-6.45	0.873	54.70	<0.001

Follow-up at four time points (baseline, 4 weeks, 6 weeks, and 3 months)

Response variable: depression score (quantitative)
Time variable: visit (baseline, 4 weeks, 6 weeks, and 3 months)
Covariate: group (Specific Auricular Acupuncture (SA), Non-Specific Auricular Acupuncture (NSA) and visit.

© 2023 de Oliveira Rodrigues DM et al. *JAMA Network Open.*

eTable 13: Blinding Assessment and Perception of Treatment Efficacy

VARIABLES	Specific Auricular Acupuncture (SA)		Non-specific Auricular Acupuncture (NSA)		p-value [a]
**Perception of Allocation Group – N (%)**					
Specific Auricular Acupuncture	14	58	14	61	0.29
Non-specific Auricular Acupuncture	6	25	2	9	
Not sure	4	17	7	30	
**Perception of Efficacy in Symptom Relief**					
Yes (treatment was effective)	13	54	12	52	0.73
No (treatment was not effective)	5	21	3	13	
Not sure	6	25	8	35	

[a] Statistical significance was calculated with Fisher's exact test.

© 2023 de Oliveira Rodrigues DM et al. *JAMA Network Open.*
