# Supplementary material for: Efficacy and Safety of Auricular Acupuncture for Depression: A Randomized Clinical Trial
Source: JAMA Netw Open. 2023 Nov 30;6(11):e2345138. doi: 10.1001/jamanetworkopen.2023.45138 (PMC10690462; doi:10.1001/jamanetworkopen.2023.45138)
Supplement: Supplement 3. — Data Sharing Statement [file jamanetwopen-e2345138-s003.pdf]

Data Sharing Statement

de Oliveira Rodrigues. Efficacy and Safety of Auricular Acupuncture for Depression. *JAMA Netw Open*. Published November 30, 2023. doi:10.1001/jamanetworkopen.2023.45138

Data
Data available: Yes
Data types: Deidentified participant data
How to access data: faisal@usp.br
When available: With publication

Supporting Documents
Document types: Statistical/analytic code, Informed consent form
How to access documents: faisal@usp.br
When available: With publication

Additional Information
Who can access the data: Anyone requesting the data
Types of analyses: For a specified purpose
Mechanisms of data availability: With investigator support
Any additional restrictions: None
